# Human pneumovirus induces IFN-dependent expression of the immune-responsive gene 1 and is inhibited by 4-octyl itaconate in human macrophages

**DOI:** 10.1093/narmme/ugag017

**Published:** 2026-03-24

**Authors:** Alix S Spahn, Kristin Rian, Alexandre Gidon, Claire Louet, Victor Boyartchuk, Trude H Flo, Marit W Anthonsen

**Affiliations:** Department of Clinical and Molecular Medicine, Norwegian University of Science and Technology (NTNU), 7006 Trondheim, Norway; Department of Clinical and Molecular Medicine, Norwegian University of Science and Technology (NTNU), 7006 Trondheim, Norway; Department of Clinical and Molecular Medicine, Norwegian University of Science and Technology (NTNU), 7006 Trondheim, Norway; Centre of Molecular Inflammation Research, Department of Clinical and Molecular Medicine, NTNU, 7491 Trondheim, Norway; Department of Clinical and Molecular Medicine, Norwegian University of Science and Technology (NTNU), 7006 Trondheim, Norway; Centre of Molecular Inflammation Research, Department of Clinical and Molecular Medicine, NTNU, 7491 Trondheim, Norway; Department of Clinical and Molecular Medicine, Norwegian University of Science and Technology (NTNU), 7006 Trondheim, Norway; Clinic of Surgery, St Olav Hospital HF, 7006 Trondheim, Norway; Department of Clinical and Molecular Medicine, Norwegian University of Science and Technology (NTNU), 7006 Trondheim, Norway; Centre of Molecular Inflammation Research, Department of Clinical and Molecular Medicine, NTNU, 7491 Trondheim, Norway; Department of Clinical and Molecular Medicine, Norwegian University of Science and Technology (NTNU), 7006 Trondheim, Norway

## Abstract

The immunometabolite itaconate, generated by immune-responsive gene 1 (IRG1/ACOD1), and its derivative 4-octyl itaconate (4OI) have been found to modulate inflammation and progression of viral infections, but their effects on the significant respiratory pathogen human metapneumovirus (HMPV) is unknown. Here, we demonstrate that HMPV induces IRG1 expression via a TANK-binding kinase 1, NF-κB-, and interferon (IFN)-dependent manner in human primary macrophages. We further show that the addition of a cell-permeable derivative of itaconate, 4OI, but not itaconate itself or its natural isomer citraconate, reduces HMPV and IFN-β levels in human macrophages. 4OI additionally activated Nrf2, while Nrf2 depletion enhanced HMPV levels, suggesting that Nrf2 mediates the antiviral effect of 4OI on HMPV. Also, we found that 4OI reduced expression of ATP-dependent citrate lyase, a lipid metabolic enzyme that supports HMPV replication. Our study suggests 4OI as a potential compound for targeting HMPV-IFN-β-driven disease and highlights Nrf2-dependent lipid reprogramming as a potential modulator of 4OI antiviral effects.

## Graphical Abstract

**Graphical Abstract:**
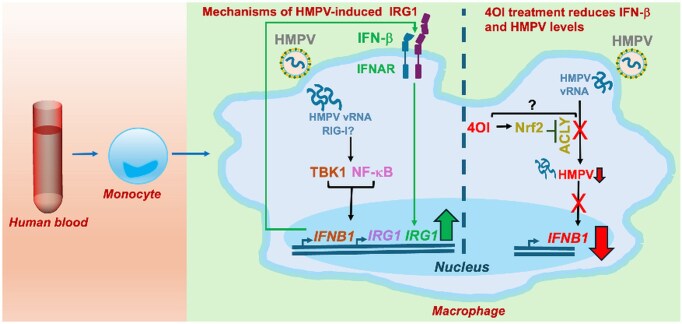

## Introduction

Human metapneumovirus (HMPV) is a negative-sense single-stranded RNA respiratory virus that belongs to the *Pneumovirus* family and is closely related to respiratory syncytial virus (RSV). HMPV may cause severe respiratory infections in young children, the elderly and immunocompromised individuals, and is associated with exacerbations of asthma and chronic obstructive pulmonary disease [1]. The host immune response fails to protect against repeated natural infections by HMPV and there are currently no treatment options or vaccines against HMPV. Hence, identification of cellular pathways and compounds that can restrict HMPV replication is a key step towards defining new strategies to treat HMPV-mediated disease.

Airway epithelial cells and innate immune cells in the lung, e.g. macrophages and dendritic cells, sense HMPV via pattern-recognition receptors like retinoic acid-inducible gene 1 (RIG-I)-like receptors (RLRs) or Toll-like receptors and respond by inducing expression of antiviral interferons (IFNs) and pro-inflammatory cytokines that are critical for limiting the viral infection [2, 3]. Importantly, excessive IFN-driven inflammation, mediated by alveolar macrophages, substantially contributes to the pathogenesis of HMPV. It has been linked to severe lung disease in humans, as demonstrated e.g. for SARS-CoV-2 and influenza virus [4–6]. Therefore, restricting induction/production of inflammatory cytokines in macrophages could represent an option to limit severe respiratory disease caused by HMPV.

The immunometabolite itaconate and itaconate derivatives have recently emerged as potential antiviral and anti-inflammatory compounds [7, 8]. Moreover, two endogenous isomers of itaconate, citraconate and mesaconate, were recently identified and proposed to have immunomodulatory capacity [9, 10]. Nevertheless, the exact nature of their cellular effects remains to be defined. The itaconate derivative 4OI has been found to exert antiviral effects against SARS-CoV-2, influenza virus, and herpes simplex virus 1, but, prior to our study, it was unknown if itaconate or its derivatives play a role in control of HMPV infections.

Itaconate is produced via decarboxylation of the Krebs cycle intermediate *cis*-aconitate by the enzyme immune-responsive gene 1 (IRG1; also known as ACOD1). Reflecting its regulatory role, IRG1 is present at very low levels in resting cells but its expression is strongly induced by inflammatory stimuli like lipopolysaccharide (LPS), particularly in macrophages, thereby stimulating anti-inflammatory responses [11–15].Knockdown of IRG1 is associated with increased immunopathology in mice infected with the pneumovirus RSV or *Mycobacterium tuberculosis* [16–18]. Moreover, the IRG1 product itaconate and particularly itaconate derivatives, such as 4OI, have emerged as negative regulators of macrophage inflammatory responses. 4OI has a stronger electrophilic potential and higher reactivity against cysteines than itaconate, e.g. in KEAP1, thereby leading to enhanced Nrf2 activation [19]. Moreover, 4OI represses macrophage type I IFN and cytokine induction in response to LPS and, as a result, reduces inflammatory aspects of human disease, e.g. sepsis and pulmonary fibrosis [7, 19]. Interestingly, 4OI also limits replication of viruses belonging to different families [20–22]. Several mechanisms have been proposed by which 4OI represses inflammation that involve the antioxidant transcription factor Nrf2 [15], the anti-inflammatory transcription factor ATF3 [13], and inhibition of the innate immune signaling proteins MAVS, IKKβ, STING, and JAK1 [16, 23, 24].

Despite substantial differences between specific mechanisms in induction of human and murine IRG1-itaconate induction and overall signal output, this immunoregulatory axis is understudied in human experimental models [25]. Macrophages are critical in virus-mediated pathogenesis, as shown for infections with HMPV, influenza virus, and SARS-CoV-2. Therefore, understanding how human viruses impact the function of the immuno-balancing protein IRG1 in human macrophages is important towards the aim of enabling our control of severe viral diseases. In this study, we set out to explore IRG1 induction and the effect of itaconate, 4OI, and citraconate in HMPV-infected primary human macrophages. We show that HMPV strongly induces IRG1 via TANK-binding kinase 1 (TBK1), NF-κB, and type I IFNs, key components of the antiviral innate immune response. Moreover, we show that 4OI, but not unmodified itaconate or citraconate, markedly attenuate IFN-β induction and reduce HMPV levels.

## Materials and methods

### Primary human monocyte-derived macrophages

Human peripheral blood monocular cells were isolated from buffy coats from anonymous human donor blood provided by the Blood Bank (St. Olav’s hospital, Trondheim) with approval by the Regional Committee for Medical and Health Research Ethics (REC Central, Norway, NO. 2009/2245). Informed consent is routinely asked for by the Blood Bank, and buffy coats are made available for research when consent is obtained. Information about the sex and age of the donors was redacted. This study used monocyte-derived macrophages (MDMs) and the cell line LLC-MK2. All cells were cultivated at 37°C under a humidified, 5% CO_2_-containing environment.

### HMPV preparation and virus infection

The clinical HMPV isolate NL/17/00 (A2; [26]) and recombinant HMPV GFP NL/1/00 (A1; [27]) were kindly provided by B. van den Hoogen (Erasmus MC, Rotterdam). HMPV strains were inoculated on LLC-MK2 cells at a multiplicity of infection (MOI) 0.01 in OptiMEM (Gibco) media containing 2% FBS (fetal bovine serum; Gibco), 50 µg/ml trypsin (Gibco), 20 µg/ml gentamicin (Gibco), and 0.7 nM glutamine (Gibco). The media was refreshed on day 4 and 6 of propagation. After 7–8 days, the virus was harvested by freeze-thawing the supernatant and cells at −80°C. The virus was purified on a 20% sucrose cushion by ultracentrifugation in swing buckets at 125 000 × *g* for 120 min at 4°C. Subsequently, the virus-containing pellet was resuspended in OptiMEM 2% FBS.

Virus titers in MDM supernatants were determined by anti-HMPV staining in Vero clone 118 cells, as previously described [28]. Briefly, supernatants were serially diluted and added to Vero cells. Seven days post-infection, cells were fixed and incubated for 1 h with monoclonal anti-HMPV antibodies (kindly provided by B. van den Hoogen, Erasmus MC, Rotterdam). After washing, cells were incubated with a FITC-conjugated rabbit anti–mouse antibody (DAKO). The endpoint infectious viral titers were calculated from quadruplicates for each individual sample using the Spearman–Kärber method and expressed as TCID_50_/ml.

MDMs were infected with HMPV at MOI 1 in the infection medium consisting of OptiMEM), 2% FBS, and 0.7 nM L-glutamine). The cells were incubated with the virus for indicated time points.

### Cell culture

Peripheral blood mononuclear cells (PBMCs) were isolated from fresh buffy coats of healthy donors obtained at the Blood Bank at St. Olav’s Hospital, Trondheim, Norway. Mononuclear cells were isolated using density gradient centrifugation (Lymphoprep™, Axis-Shield Point of Care). Isolated PBMCs were seeded in RPMI 1640 medium supplemented with 10% human serum (the Blood Bank, St. Olav’s Hospital) and 0.34 mM L-glutamine (Gibco). After 1 h at 37°C and 5% CO_2_ monocytes were separated from PBMCs by plastic adhesion followed by three washing steps with Hanks’ balanced salt solution (Gibco). Cells were differentiated to MDMs for 7–10 days in RPMI 1640 culture medium supplemented with 10% human serum, 0.34 mM L-glutamine, and 10 ng/ml macrophage colony stimulating factor (M-CSF; Sigma). Media was replaced 1 day prior experiments to M-CSF-free culture medium or infection medium.

### Compound treatments

LPS was sonicated for 30 s at 37°C followed by 3 × 3 s vigorous shaking. Subsequently, MDMs were stimulated with 500 ng/ml or 10 µg/ml LPS (Sigma) supplemented in culture medium without M-CSF. High molecular weight poly IC (Invitrogen) was transfected using Lipofectamine^TM^ 2000 (Invitrogen) according to the manufacturer’s instructions. Inhibition with S-Ruxolitinib (Merck) or GSK 872 (GlaxoSmithKline) was performed 1 h before infection with HMPV, whereas BMS-303141 (MedChemExpress) was preincubated for 2 h. The IFNAR neutralizing antibody (nIFNAR, clone MMHAR-2) was purchased from PBL; 5 or 10 µg/ml were incubated for 30 min before infection with HMPV. Stimulation with 100 U/ml recombinant IFN-β (Preprotech) was conducted for 3 or 24 h. The compounds itaconate (ITA), citraconate (CIT), 4-octyl itaconate (4OI), and dimethylsulfoxide (DMSO) were purchased from Sigma. ITA and CIT were dissolved in phosphate-buffered saline (PBS) and pH-adjusted to pH 7.2. 4OI was dissolved in DMSO to a stock concentration of 250 mM. The four compounds were supplemented into infection media 12 h before infection with the virus.

### RNA interference in MDMs

Transfection of MDMs with small interfering RNA (siRNA) was performed with Lipofectamine^TM^ RNAiMAX (Invitrogen) following the manufacturer’s protocol. MDMs were treated siRNA against IRF1, RIPK3, Nrf2 (Dharmacon, pooled ON-TARGETplus), and RelA/p65 (Ambion) or a non-targeting siRNA (Qiagen, AllStar negative control). The final siRNA concentration was 10 nM, if not otherwise stated. The transfection mix was incubated on MDMs for 24 h at 37°C. This process was then repeated, and the transfection mix was replaced with a fresh one. After the completion of the second siRNA transfection, the medium was changed to the appropriate treatment media. MDMs were allowed to rest overnight before further treatments. Gene knockdown was determined by reverse transcription quantitative polymerase chain reaction (RT-qPCR). A gene knockdown >50% was considered efficient.

### Immunoblotting

Whole cell lysates were prepared in lysis buffer (50 mM Tris, 150 mM NaCl, 10% glycerol, 1% Triton X-100, and 2 mM ethylenediaminetetraacetic acid) containing phosphatase and protease inhibitor cocktails. Protein extracts were separated by NuPAGE Bis-Tris gels (Thermo Fisher Scientific). Dry blotting was performed using iBlot Gel Transfer stacks Nitrocellulose Mini kit and iBlot® machine (Invitrogen). Membranes were blocked for 1 h with lntercept Blocking Buffer (LI-COR Biosciences) and washed prior to incubation with primary antibodies. The primary human antibodies IRG1, IRF1, RIPK3, NF-κB p65, phospho-STAT1 (Tyr701), STAT1, and Nrf2 were purchased from Cell Signalling Technology. The following antibodies were purchased from the indicated suppliers: HMPV-N (Abcam) and HO-1 (Enzo Life Science). Secondary antibodies (IRDye 800CW Goat anti-Mouse, IRDye 800CW Goat anti-Rabbit, IRDye 680RD Goat anti-Mouse, IRDye 680RD Goat anti-Rabbit) were purchased from LI-COR Biosciences. In Figs 3–5, the immunoblots were reblotted and used for all figures and show the same housekeeping gene, GAPDH, respectively to the treatment. LI-COR Odyssey imager was used as the scanning system and for the determination of band intensities.

### RNA extraction, complementary DNA synthesis, and qRT-PCR

Cells were lysed in RTL lysis buffer (Qiagen) containing 1% β-mercaptoethanol and frozen at −20°C. Total RNA was extracted with the RNeasy Mini Kit (Qiagen) according to the manufacturer’s instructions, including a 15-min DNase I (Qiagen) digest. The RNA was synthesized to complementary DNA using the qScript kit (Quanta) following the manufacturer’s protocol. Quantitative real-time PCR (qRT-PCR) was performed using the Perfecta SYBR Green reaction mix (Quanta) and a StepOnePlus instrument (Life Technologies). Reverse transcripts were amplified with the primer sequences listed in Supplementary Table S1 using the temperature profile: 95°C for 20 s, 40 cycles at 95°C for 3 s, and 60°C for 30 s. Fold changes were estimated using the ΔΔC_T_ technique after data normalization to GAPDH [29]. Relative messenger RNA (mRNA) concentrations are expressed in fold change compared to the uninfected group. The fold change of the HMPV N-gene expression was calculated relative to an 1 h infected sample.

### Neutral lipid staining and imaging

MDMs were cultivated on glass-bottomed 96-well plates and differentiated for 12 days. The cells were either left uninfected or challenged with GFP-expressing recombinant HMPV at MOI 1 for 24 h. Subsequently, MDMs were fixed in 4% paraformaldehyde for 10 min at room temperature. Neutral lipids were stained with HCS LipidTOX™ Deep Red (Thermo Fisher Scientific) following the manufacturer’s instructions.

MDMs were imaged with a Zeiss LSM880 confocal microscope with a Plan-Apochromat 20× numerical aperture objective of 0.8. Emissions were collected using GaAsP detectors. GFP was excited with a 488-nm argon laser, and emissions were detected through a 505–550 window. HCS LipidTOX™ Deep Red was excited with a 633-nm HeNe diode laser, and emissions were collected through a 645–700-nm window. Images were acquired from six fields of view per condition, yielding ~2000 macrophages total per condition. Images were analysed with ImageJ (Fiji).

### Quantification and statistical analysis

Statistical details are given in the figure legends. Unless otherwise specified, data are presented as individual donors (*n*), obtained from 3 to 6 independent donors. Statistical analysis was performed in GraphPad Prism [Version 9.1.2. (266)]. For single comparison a paired Student’s *t*-test with Tukey post-hoc test was chosen, whereas multiple comparisons were analysed by paired one-way analysis of variance (ANOVA) with Tukey post-hoc test. Differences were expressed in SEM and considered significant when *P < .05; **P < .01; ***P < .001; ****P < .0001 or ns, not significant.

## Results

### HMPV induction of IRG1 in human macrophages is dependent on type I IFN

**Figure 1.**
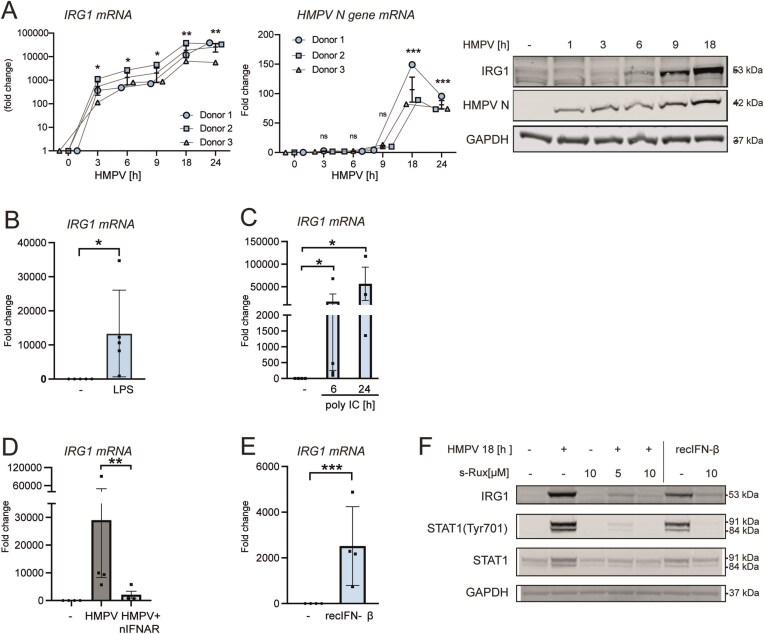
HMPV induction of IRG1 in human macrophages is dependent on type I IFN. (**A**) MDMs were infected with HMPV for the indicated time points. HMPV N gene or *IRG1* mRNA levels were determined by qRT-PCR analysis (*n* = 3), while protein levels of IRG1, HMPV N, and GAPDH were analysed by immunoblotting (*n* = 2). IRG1 protein levels shown are for the same donor sample used in Fig. 1D of [31], thus panels showing HMPV N and GAPDH levels being identical to the ones shown [31]. (**B**) MDMs were treated with 500 ng/ml LPS for 2 h. *IRG1* mRNA levels were assessed by qRT-PCR and normalized to untreated (−) control (*n* = 5). (**C**) MDMs were transfected with 10 μg/ml poly IC for 6 or 24 h. *IRG1* mRNA levels were assessed by qRT-PCR and normalized to untreated (−) cells (*n* ≥ 2). (**D**) MDMs were pre-incubated (30 min) or not with 10 μg/ml of neutralizing IFNAR antibody before infection with HMPV for 24 h. *IRG1* mRNA expression was determined using qRT-PCR and normalized to untreated (−) cells (*n* = 4). (**E**) MDMs were treated with 100 U/ml recIFN-β for 24 h. *IRG1* mRNA expression was determined using qRT-PCR and normalized to untreated (−) cells (*n* = 4). (**F**) MDMs were preincubated with the JAK1/JAK2 inhibitor ruxolitinib (5 or 10 μM) prior to infection with HMPV for 18 h or treatment with IFN-β for 3 h. Protein levels in whole cell lysates were analysed for IRG1, STAT1 (Tyr701), STAT1, and GAPDH via immunoblot (*n* = 2).

We first assessed whether HMPV infection affected IRG1 levels in human macrophages. Human MDMs were infected with HMPV for 1–24 h prior to analysis of IRG1 mRNA by qRT-PCR or quantification of protein levels by immunoblot. We found that IRG1-levels in uninfected human MDMs were low, and that both IRG1 mRNA and protein levels were highly induced by HMPV infection in a time-dependent manner (Fig. 1A). IRG1 protein levels largely followed the increase in HMPV N mRNA and protein (Fig. 1A). At the timepoints examined, HMPV induced IRG1 mRNA in MDMs to similar levels as the common pathogen recognition receptor ligands LPS (TLR4 ligand) and polyIC (endosomal TLR3 and cytosolic RLR ligands) (Fig. 1B and C). These results show that in human macrophages, HMPV infection results in a marked upregulation of IRG1.

IFN-β enhances IRG1 induction by LPS [15]. Our previous studies showed that HMPV infection of MDMs strongly induced expression of type I IFNs from 18 h of infection onwards [30, 31]. To determine if type I IFNs contribute to HMPV-stimulated IRG1 induction, human MDMs were incubated with anti-IFNAR neutralizing antibodies prior to infection with HMPV. Blocking of IFNAR strongly reduced HMPV-driven IRG1 mRNA upregulation (Fig. 1D). We also confirmed that treatment with recombinant IFN-β stimulated IRG1 mRNA expression in human MDMs (Fig. 1E and F). Typically type I IFNs induce gene expression via IFNAR-JAK1-mediated STAT1 phosphorylation [32]. Ruxolitinib is a potent inhibitor of STAT1-mediated gene induction [32–34]. We therefore chose to test the effect of the Janus family kinases (JAK1 and JAK2) inhibitor ruxolitinib on IRG1 induction in response to HMPV infection. We found that preincubation with ruxolitinib impaired HMPV- and IFN-β-stimulated increase of IRG1 protein (Fig. 1F), showing that IFNAR signaling is critical to IRG1 induction by HMPV in human macrophages.

### TBK1 and NF-κB positively regulate IRG1 levels in HMPV-infected human macrophages

**Figure 2.**
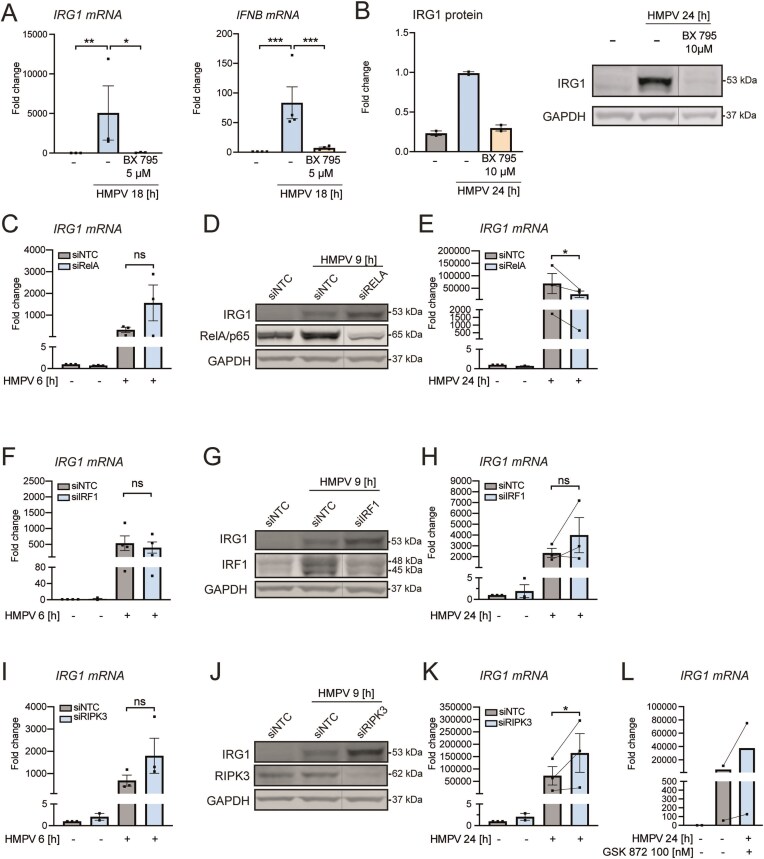
TBK1 and NF-κB positively regulate IRG1 levels in HMPV-infected human macrophages. MDMs were incubated with 5 or 10 μM of the TBK1 inhibitor BX795 for 30 min before infection with HMPV for 24 h. (**A**) *IRG1* and *IFN-β* mRNA levels (*n* ≥ 3) were analysed by qRT-PCR (left panels), while in panel (**B**) IRG1 and GAPDH protein levels were determined by immunoblotting, quantified, and presented with SD relative to uninfected cells treated with DMSO (*n* = 2; right panel). (C–K) MDMs were transfected with siRNAs targeting the NF-κB subunit *RELA* (C–E), *IRF1* (F–H), *RIPK3* (I–K), or control siRNA (siNTC) before infection with HMPV for 6, 9, or 24 h and analysis of *IRG1* mRNA levels by qRT-PCR or IRG1, RelA/p65, IRF1, RIPK3, or GAPDH protein levels by immunoblotting. *IRG1* mRNA levels were assessed relative to siNTC-transfected, uninfected MDMs for all experiments. (**C, E**) *IRG1* mRNA after 6 h (*n* = 3) or 24 h HMPV (*n* = 3). (**D**) Protein levels of IRG1, RelA/p65, and GAPDH (*n* = 3) after 9 h HMPV. (**F, H**) *IRG1* mRNA after 6 h (*n* = 4) or 24 h of infection (*n* = 3). (**G**) Protein levels of IRG1, IRF1, and GAPDH (*n* = 3) after 9 h HMPV. (**I, K**) *IRG1* mRNA after 6 h (*n* = 3) or 24 h of infection (*n* = 3). (**J**) Protein levels of IRG1, IRF1, and GAPDH (*n* = 3) after 9 h HMPV. (**L**) MDMs were incubated with 100 nM of the RIPK3 inhibitor GSK 872 for 1 h before infection with HMPV for 24 h. *IRG1* mRNA levels were normalized relative to uninfected cells treated with DMSO (*n* = 2). Single comparison between control (NTC) siRNA and target-siRNA conditions were calculated using paired *t*-test with Tukey post-hoc test. *P < .05, **P < .01, ***P < .001, ****P < .0001; ns = non-significant. See also Supplementary Fig. S1.

Bourner *et al.* [35] recently reported that IRG1/itaconate was induced by multiple innate immune signaling pathways, of which many, but not all, required TBK1 signaling. Hence, we tested the effect of the TBK1 inhibitor BX795 and found that TBK1 inhibition in human macrophages strongly reduced IRG1 induction by HMPV at the mRNA (Fig. 2A) and protein level (Fig. 2B), with similar effects at 5 and 10 μM BX795 (Fig. 2, data not shown). HMPV-induced IFN-β mRNA was also strongly reduced after BX795 treatment (Fig. 2A, middle panel).

We have previously shown that HMPV activates NF-κB signaling in human MDMs and that TBK1 regulates IFN induction in HMPV-infected cells [31]. Also, the NF-κB pathway has been reported to mediate IRG1 induction in murine models of *M. tuberculosis* infection [36]. To address the question if the NF-κB pathway contributes to HMPV-induced IRG1 expression, we transfected MDMs with siRNA against *RELA*, which encodes the NF-κB subunit p65. We first confirmed that treatment with siRELA reduced *RELA* expression levels (Supplementary Fig. S1). Subsequently, we found that silencing of RelA/p65 did not alter IRG1 gene- and protein expression at early time points (3, 6, and 9 h) of HMPV infection, whereas IRG1 expression was decreased in *RELA*-depleted cells at later timepoints of infection, showing the same inhibitory trend in all donors (24 h; Fig. 2C–E and Supplementary Fig. S1). The variation in HMPV-induced levels of IRG1 (in siNTC-treated MDMs) illustrates that the magnitude of IRG1 induction varies among human donors, similarly to as previously shown for other inflammatory stimuli [16, 37].

We have previously found that IRF1 contributed to IRG1 induction by *Mycobacterium avium* [38]. To evaluate the involvement of IRF1 to HMPV-stimulated IRG1 induction, we performed siRNA-mediated knockdown of IRF1 in MDMs. In our experiments, the knockdown efficiency was ~80% compared to cells treated with non-targeting control siRNA (siNTC; Supplementary Fig. S1). In contrast to what we observed for *Mycobacterium avium* infection [38], we found that IRF1-depletion did not significantly reduce neither IRG1 mRNA nor protein levels after HMPV infection for 3–24 h (Fig. 2F–H and Supplementary Fig. S1). It is possible that, similarly to many documented IRF-driven signaling responses, individual IRFs may be redundant [39, 40] and that additional IRFs may contribute to HMPV-stimulated IRG1 induction in IRF1-depleted cells. Nevertheless, based on ours and others’ results [35, 36], it appears that TBK1, NF-κB, and type I IFN signaling are important regulators of IRG1 levels after exposure to HMPV and other pathogen-associated stimuli.

A recent study suggested that IRG1 levels are linked to RIPK3 (Receptor-Interacting serine/threonine-protein kinase 3) in Zika virus–infected mouse neurons [41]. To determine if RIPK3 contributes to HMPV-stimulated IRG1 expression, we silenced RIPK3 in human MDMs using siRNA (Supplementary Fig. S1). We found that RIPK3 depletion led to significant increase in IRG1 mRNA levels at 3 and 24 h post-infection and a corresponding increase in IRG1 protein levels (Fig. 2I–K and Supplementary Fig. S1). Moreover, preincubation of MDMs with the RIPK3 inhibitor GSK'872 showed a similar tendency with a modest increase in HMPV-stimulated IRG1 mRNA levels (Fig. 2L), hence differing from the effect of RIPK3 in Zika virus–infected mouse neurons [41]. Taken together, our data show that TBK1, NF-κB, and type I IFN signaling are important regulators of IRG1 levels in HMPV-infected MDMs.

### Depletion of IRG1 in macrophages changes HMPV levels in donor-specific manner

Following our discovery of strong induction of IRG1 expression with HMPV infection, we next determined if IRG1 depletion impacted HMPV burdens in MDMs. Treatment with siIRG1 reduced IRG1 mRNA levels below 35% (Supplementary Fig. S2). However, unexpectedly, we found that depleting IRG1 variably increased or decreased HMPV levels in MDMs derived from different human donors, hence displaying donor-dependent differences (Supplementary Fig. S2). In this regard, IRG1 regulation and expression have indeed been found to depend on age and sex [20, 42, 43]. Although this is an important issue to address experimentally, the information about sex or age of individual blood donors is not disclosed to us. We therefore were unable to correlate the variation in IRG1-silencing effects on HMPV MDM cell burdens to these factors.

### Exogenously added 4OI but not itaconate or citraconate reduces HMPV levels

**Figure 3.**
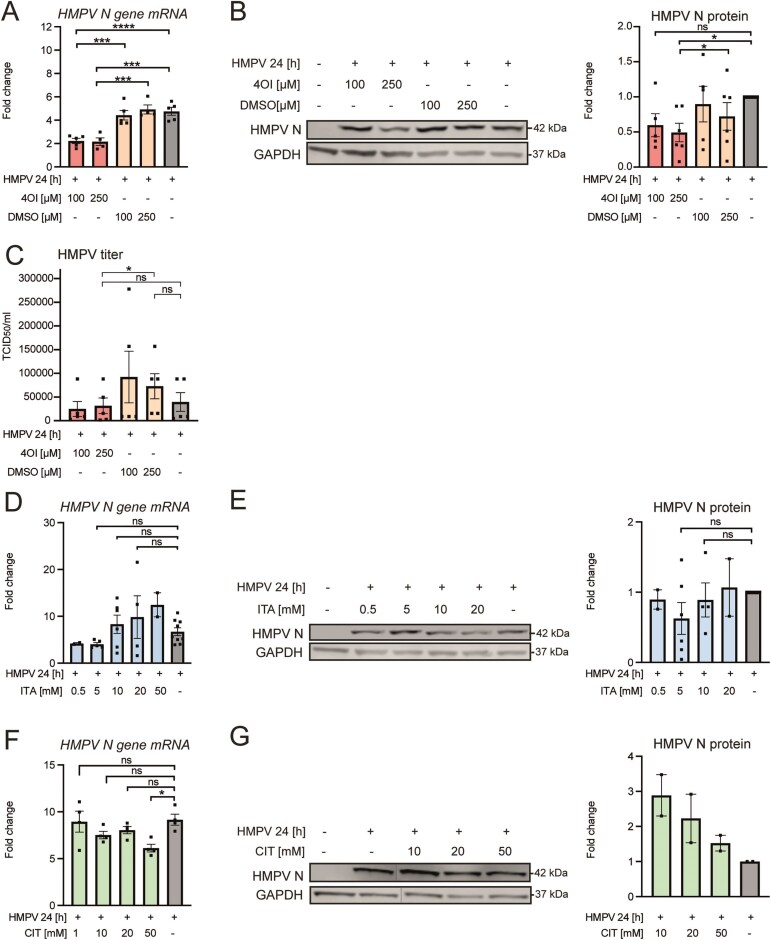
Exogenously added 4OI, but not itaconate or citraconate, reduces HMPV levels in human MDMs. (A–C) MDMs were preincubated with 100 or 250 µM 4OI or its vehicle, DMSO, prior to HMPV infection for 24 h. Levels of *HMPV N-gene* mRNA were quantified by qRT-PCR (**A**; *n* ≥ 5), HMPV N and GAPDH protein levels were analysed by immunoblotting (**B**; *n* ≥ 5), while infectious HMPV/HMPV titers in MDM supernatants was determined as TCID_50_/ml (**C**; *n* = 5). (D, E) MDMs were treated with itaconate (ITA; 0.5, 5, 10, and 20 mM) prior to HMPV infection for 24 h. Levels of *HMPV N-gene* mRNA were quantified by qRT-PCR (**D**; *n* ≥ 2), while HMPV N and GAPDH proteins were analysed by immunoblotting (**E**; *n* ≥ 2). (F, G) MDMs were treated with citraconate (CIT; 1, 10, 20, and 50 mM) prior to HMPV infection for 24 h. Levels of the *HMPV N-gene* mRNA were quantified by qRT-PCR (**F**; *n* = 4), while HMPV N and GAPDH protein levels were analysed by immunoblotting (**G**; *n* = 2). Protein levels were quantified by normalizing band intensities against GAPDH and expressed as fold change compared to untreated (i.e. no itaconate variant) but infected MDMs. Multiple comparisons were analysed by a paired onw-way ANOVA with Tukey post-hoc test. Significance was ranked as *P < .05; **P < .01; ***P < .001; ****P < .0001, and ns, not significant.

As an alternative strategy for evaluating the impact of IRG1-derived itaconate on HMPV levels, we examined the effect of exogenously supplemented itaconate, its cell-permeable derivative 4OI, and the natural isomer citraconate on HMPV-infected macrophages. MDMs were pretreated with different concentrations of 4OI or its vehicle, itaconate, or citraconate before infection with HMPV for 24 h and measurement of HMPV-N mRNA or protein levels by qRT-PCR or immunoblot analysis, respectively. Cytotoxicity of the itaconate compounds in MDMs was tested in lactate dehydrogenase (LDH) leakage assay, which showed that none of the applied concentrations exceeded 3% toxicity after a 48-h incubation period (data not shown). We found that pretreatment with both 100 and 250 μM 4OI significantly reduced HMPV-N mRNA and protein levels in infected MDMs compared to the vehicle control (Fig. 3A and B). To determine if 4OI affected HMPV infectivity, we determined HMPV titers in supernatants of MDMs treated with DMSO or 4OI prior to HMPV infection using an end-point dilution assay expressing titers as TCID_50_/mL. The results show that 250 μM 4OI pretreatment significantly suppressed HMPV titers, whereas the effect of 100 μM 4OI was not significant (Fig. 3C). In contrast, in similarly infected macrophages, itaconate pre-treatment did not significantly affect HMPV-N mRNA or protein levels (Fig. 3D and E), while citraconate led to significant reduction only of HMPV-N mRNA levels only at the highest tested concentration (Fig. 3F and G).

### 4OI, itaconate, and citraconate differ in regulating IRG1 and the IFN-β response upon HMPV infection

**Figure 4.**
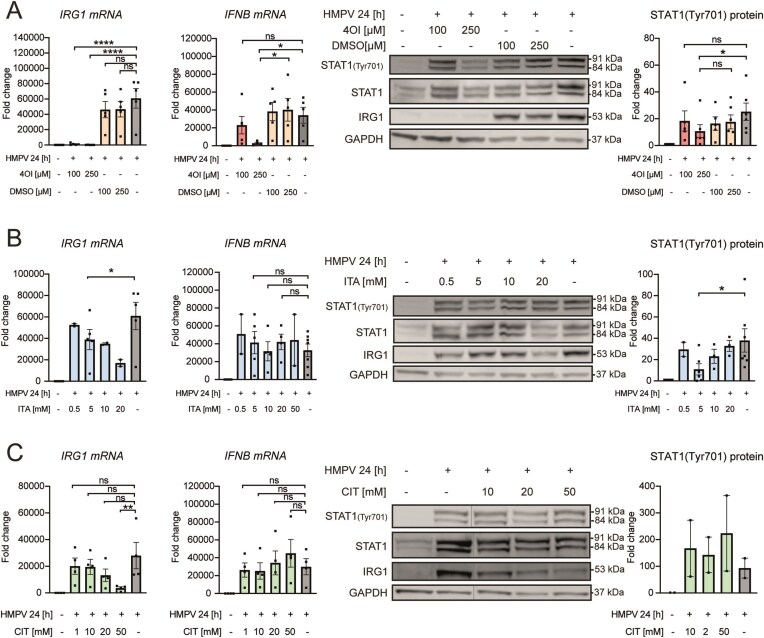
4OI, itaconate, and citraconate differ in regulating IRG1 and the IFN-β response upon HMPV infection. MDMs were preincubated with (**A**) 100 and 250 µM of 4OI or DMSO, (**B**) 0.5, 5, 10, and 20 mM of itaconate, and (**C**) 1, 10, 20, and 50 mM of citraconate prior to infection with HMPV for 24 h. Levels of *IRG1* and *IFNB* mRNA (left panels) were quantified by qRT-PCR. The expression of STAT1 (Tyr701), STAT1, IRG1, and GAPDH protein (right panels) was analysed using immunoblotting of whole-cell lysates and quantification STAT1 (Tyr701) expression. (**A**) *n* ≥ 5 for mRNA and protein; (**B**) *n* ≥ 2 for mRNA and protein; and (**C**) *n* = 4 for mRNA and *n* = 2 for protein. *IFNB* and *IRG1* mRNA levels were assessed relative to untreated, uninfected MDMs for all experiments. Protein levels were quantified by normalization of band intensities against GAPDH and was expressed as fold change compared to untreated, uninfected MDMs. Multiple comparisons were analysed by a paired onw-way ANOVA with Tukey post-hoc test. Significance was ranked as *P < .05; **P < .01; ***P < .001; ****P < .0001, and ns, not significant.

To identify host immunomodulatory factors controlling observed differences in HMPV burdens in infected MDMs, we next examined evaluated how tested itaconate species impact levels of the IRG1 and IFN-β in HMPV-infected cells. All three compounds reduced IRG1 mRNA and protein levels in HMPV-infected macrophages, with 4OI having the strongest effect (Fig. 4 left panels and immunoblots). As HMPV-induced IRG1 expression is IFN-dependent, we next evaluated if the observed reduction of IRG1 levels following treatments with itaconate and itaconate derivatives was associated with reduced IFN-β induction. Indeed, 4OI strongly inhibited induction of IFN-β mRNA by HMPV, while itaconate and citraconate did not significantly change IFN-β levels in HMPV-infected cells (Fig. 4). As STAT1 is essential for the biological effects of type I IFNs, is activated via IFNAR-JAK1 signaling [32, 44], and its phosphorylation has previously been found to correlate to IFN-β levels [45, 46], we monitored phosphorylated STAT1 levels. We found that, depending on the concentrations, 4OI and itaconate suppressed Tyr701 STAT1 phosphorylation a key step in IFN-β signaling, while citraconate treatment did not significantly affect STAT1 phosphorylation (Fig. 4, right panels). In conclusion, the 4OI-mediated reduction of IRG1 levels was associated with markedly reduced IFN-β induction and signaling. In contrast, although both itaconate and citraconate dose-dependently reduced IRG1 levels induced by HMPV (though not as potently as 4OI), only itaconate reduced STAT1 phosphorylation (Fig. 4). These results are similar to differential effects of itaconate species that were observed in influenza virus-infected cells [20] and suggest that IFN-independent mechanisms could contribute to IRG1 expression, e.g. via cytokine-dependent mechanisms as we previously found for *Mycobacterium avium* [38].

### The Nrf2 pathway is induced by 4OI, but not by itaconate or citraconate, and limits HMPV in human macrophages

**Figure 5.**
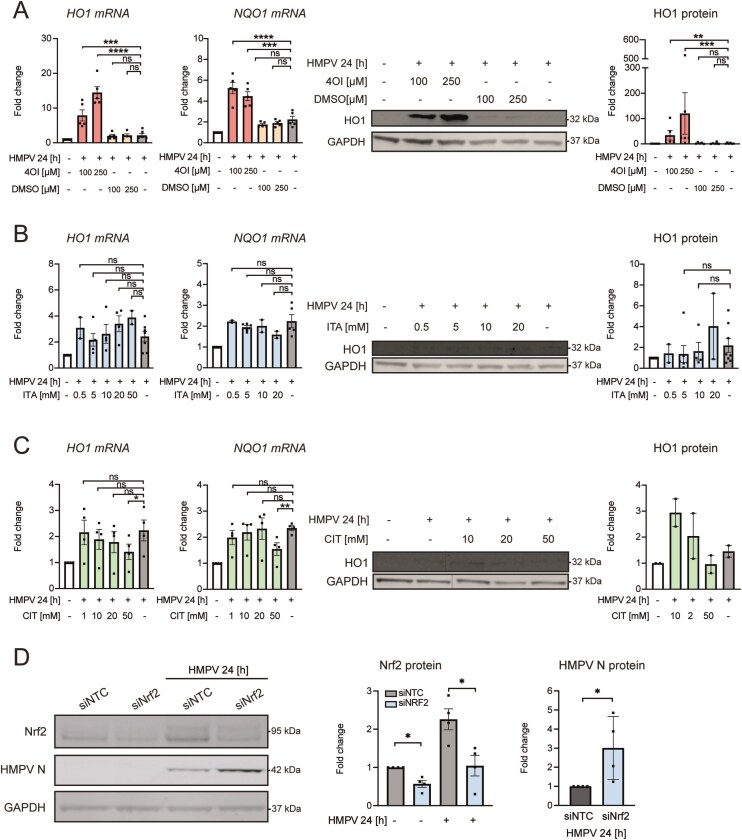
The Nrf2 pathway is induced by 4OI, but not by itaconate or citraconate, and limits HMPV in human macrophages. MDMs were preincubated with (**A**) 100 and 250 µM of 4OI or DMSO, (**B**) 0.5, 5, 10, and 20 mM of itaconate, and (**C**) 1, 10, 20, and 50 mM of citraconate prior to infection with HMPV for 24 h. (A–C) Levels of *HO1* and *NQO1* mRNA were quantified by qRT-PCR. The expression of HO1 and GAPDH protein was analysed using immunoblotting of whole cell lysates. (**A**) *n* ≥ 5 for mRNA and protein; (**B**) *n* ≥ 2 for mRNA and protein; (**C**) *n* = 4 for mRNA and *n* = 2 for protein. *HO1* and *NQO1* mRNA levels were assessed relative to untreated, uninfected MDMs for all experiments. HO1 protein levels were quantified by normalization of band intensities against GAPDH and expressed as fold change compared to untreated, infected MDMs. (**D**) MDMs were transfected with 20 nM *NFE2L2* (Nrf2) siRNA or siNTC and infected with HMPV for 24 h. Nrf2, HMPV N protein, and GAPDH were analysed by immunoblotting of whole cell lysates and quantified (*n* = 4). Nrf2 protein level is presented as fold change relative to siNTC-treated uninfected MDMs (left panel), while HMPV N-protein is presented as relative to siNTC-treated HMPV-infected MDMs (right panel). In panels (A–C), multiple comparisons were analysed by a paired onw-way ANOVA with Tukey post-hoc test, in panel (D) unpaired Student’s *t*- test. Significance was ranked as *P < .05; **P < .01; ***P < .001; ****P < .0001 and ns, not significant.

4OI alkylates and reduces Keap1 levels, thereby activating the Nrf2-pathway, which suppresses inflammation and modulates virus replication [15, 21]. Hence, we evaluated the effect of itaconate compounds on Nrf2 activation by establishing the impact of 4OI, itaconate, and citraconate on expression of the Nrf2-regulated genes HO-1 and NQO1 in HMPV-infected macrophages. We found that while 4OI strongly induced NQO1 and HO-1 mRNA and HO-1 protein levels, itaconate and citraconate had only moderate or no effect on NQO1 land HO-1 levels (Fig. 5A–C; dark blots in Fig. 5B and C are due to HO-1 protein levels below/at the detection limit). The Nrf2 pathway, activated by 4OI, can limit virus levels, e.g. SARS-CoV-2 [21], and we next explored if siRNA-mediated reduction of Nrf2 (*NFE2L2*) affected HMPV levels. Nrf2 was significantly reduced after siNrf2-transfection (Fig. 5D), and knockdown of Nrf2 led to a significant increase in HMPV N-protein levels in infected MDMs (Fig. 5D). Collectively, these results show that only 4OI limits HMPV, suppresses IFN-β levels, and activates the Nrf2-pathway in human macrophages. Hence, it is possible that 4OI suppresses HMPV levels through its induction of the Nrf2 pathway.

### 4OI reduces expression of ATP-dependent citrate lyase that is required for HMPV replication

**Figure 6.**
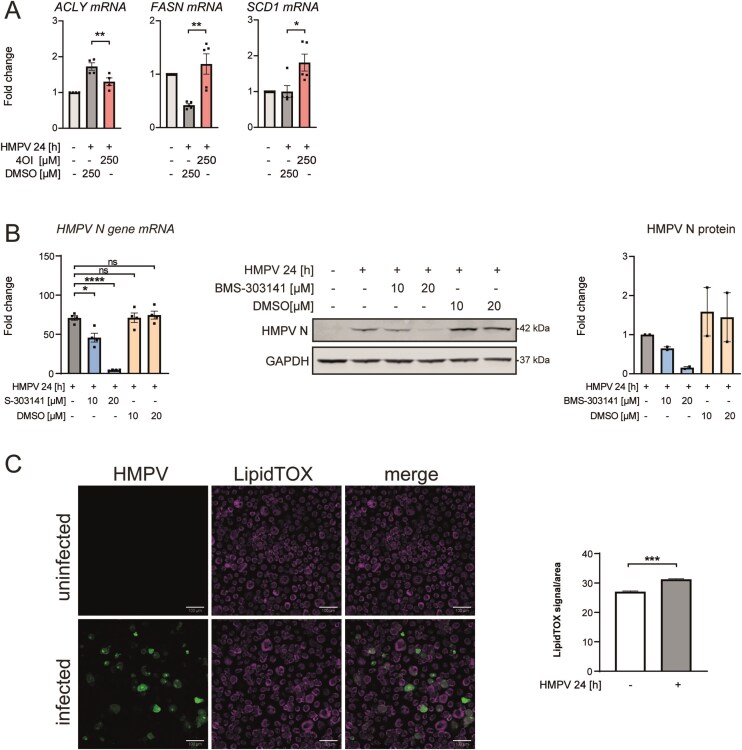
4OI reduces expression of ATP-dependent citrate lyase required for HMPV replication. (**A**) 4OI reduces ATP-dependent citrate lyase (*ACLY)* expression. MDMs were treated with 4OI (250 µM) or DMSO prior to infection with HMPV for 24 h. Expression levels of *ACLY, FASN*, and *SCD1* mRNA were quantified relative to untreated, uninfected MDMs via qRT-PCR (*n* ≥ 4). (**B**) ACLY inhibition reduces HMPV levels. MDMs were pretreated with 10 or 20 µM of the ACLY inhibitor BMS-303141 or DMSO prior to infection with HMPV for 24 h. *HMPV N-gene* mRNA was determined by qRT-PCR (*n* = 4). Protein expression of HMPV N and GAPDH was analysed via immunoblotting of whole cell lysates (*n* = 2). Protein levels were quantified by normalizing of band intensities against GAPDH and expressed as fold change compared to siNTC-transfected, infected MDMs. Multiple comparisons were analysed by a paired one-way ANOVA with Tukey post-hoc test. Significance was ranked as *P < .05; **P < .01; ***P < .001; ****P < .0001 and ns, not significant. (**C**) HMPV stimulates increased level of neutral lipids in infected MDMs. MDMs were left uninfected (−) or infected with a MOI 1 of GFP-expressing recombinant HMPV for 24 h. HCS LipidTOX™ Deep Red neutral lipid stain was used to monitor lipids by confocal microscopy. Left panels: Representative images for each treatment showing HCS LipidTOX™ Deep Red (magenta) and HMPV (green). Right panel: Quantification of the LipidTOX™ signal per area. Signal was quantified from z-stacks of six fields of view per condition using the 20× numerical aperture yielding to ~2000 cells per condition and normalized to the area. Scalebar is adjusted to 100 µm (*n* = 1). The data were assessed for normality and then compared with a non-parametric, two-tailed Mann–Whitney test. Significance was ranked as *P < .05; **P < .01; ***P < .001; ****P < .0001, and ns, not significant.

Interestingly, recent reports have shown that endogenous IRG1 and 4OI-mediated Nrf2 activation modulate cellular lipid metabolism. In murine models, 4OI treatment attenuated atherosclerosis development that is induced by a high-cholesterol diet [43, 47–49]. Nrf2 is known to control transcriptional regulation of *de novo* lipogenic enzymes [50]. Hence, we chose to determine the expression levels of ATP citrate lyase (ACLY), fatty acid synthase (FASN), and stearoyl-CoA 9-desaturase (SCD1) in 4OI-treated HMPV-infected cells. These genes were selected since Nrf2 has been found to negatively regulate ACLY [51] while positively regulating FASN and SCD1 levels [52]. Consistently, we found that 4OI-treatment of HMPV-infected cells significantly reduced ACLY mRNA levels and increased FASN and SCD1 mRNA levels (Fig. 6A), which is consistent with the reported effect of Nrf2 activation on these genes (as 4OI stimulates Nrf2 activation) and suggests that 4OI may affect lipid biosynthesis in human macrophages.

Since 4OI lowered both HMPV and ACLY levels in HMPV-infected MDMs, we next tested if inhibiting ACLY function by a chemical inhibitor impacted HMPV replication. Indeed, we found that pretreating MDMs with the ACLY inhibitor BMS-303141, known to reduce lipid biosynthesis [53], strongly suppressed HMPV mRNA and protein levels in a dose-dependent manner (Fig. 6B). This observation suggests that ACLY activity and lipid biosynthesis in general facilitate HMPV replication. Interestingly, using LipidTox staining and confocal microscopy imaging, we observed that HMPV infection of MDMs led to a significant increase in intracellular neutral lipids (Fig. 6C), further supporting that lipid biosynthesis contributes to HMPV replication. Indeed, many viruses depend on lipid biosynthesis for their replication, and targeting this lipidomic reprogramming has been suggested as a target for developing broad-spectrum antiviral strategies [54].

Taken together, our results show that 4OI-activates Nrf2-signaling and reduces ACLY mRNA levels, which may be linked to the antiviral effect of 4OI against HMPV.

## Discussion

The IRG1-generated immunometabolite itaconate and its derivatives have emerged as compounds with significant anti-inflammatory and antiviral properties. A broad range of viruses—influenza virus, SARS-CoV-2, HSV-1/2, vaccinia virus, and Zika virus—have been shown to be inhibited by 4OI, a cell permeable derivative of itaconate [20–22]. However, to date, the effects of itaconate and its derivatives on the critical human airway pathogen HMPV were not known. Macrophages, major producers of innate immune cytokines, together with excessive type I IFN induction cause severe HMPV-mediated pathogenesis [6, 55]. In our study we report that in human macrophages HMPV stimulates IRG1 expression and that addition of 4OI suppresses both IFN-β induction and HMPV burden.

Despite the recognized importance of the IRG1-itaconate axis for regulation of human inflammatory diseases, IRG1 transcriptional regulation remains largely unknown. To date, most studies of the IRG1-itaconate regulatory axis have been done in LPS-treated mouse macrophages. Considering that human and mouse macrophages differ with regard to levels of intracellular itaconic acid concentration induced and the effect that IRG1-itaconate has on inflammatory cytokine induction [35, 37], data generated from human macrophages is urgently needed to devise specific strategies for targeting onset of inflammation. We found that in HMPV-infected human macrophages TBK1, NF-κB, and type I IFN signaling regulate HMPV-mediated IRG1 induction, similarly to their previously documented involvement in LPS- or *Mycobacterium avium*-driven IRG1 induction [15, 38]. In contrast to our findings with *M. avium* [38] IRF1 depletion did not reduce HMPV-stimulated IRG1 expression. It is possible that, similarly to many documented IRF-driven signaling responses, individual IRFs may be redundant [39, 40]. Likewise, we did not observe reduced IRG1 expression in HMPV-infected MDMs after RIPK3 depletion, which differs from observations in Zika virus-infected mouse neurons [41]. The reason for this difference remains to be established, but as RIPK3 is strongly linked to cellular metabolism [56] and neurons have distinct metabolic profiles compared to macrophages, this could affect the impact of RIPK3 on IRG1 induction. As IRG1 suppresses pro-inflammatory cytokine levels, our findings that type I IFNs stimulate IRG1 levels in virus-infected cells is relevant considering that appropriate timing of IFN induction is critical to avoid hyperinflammation and systemic cytokine storm, as observed in severe SARS-CoV-2 or influenza virus infection [4, 5]. It would therefore be interesting to determine if IRG1 induction, known to restrain hyperinflammation e.g. in sepsis [37], is delayed or reduced in individuals with severe viral disease such as COVID-19, hence being a possible contributor to excessive inflammation in the disease. Indeed, low levels of plasma itaconate have been associated with excessive inflammation in SARS-CoV-2-infected individuals [57]. Also, Bourner *et al.* [35] recently identified that in the human macrophage cell line THP1, IRG1 was critical to avoid high levels of proinflammatory cytokines induced via multiple pathogen recognition receptors, including RIG-I-like receptors, which are likely implicated in recognizing HMPV in macrophages [1, 58]. Another clinically relevant aspect of type I IFNs inducing IRG1 is that IFN-β is used as standard-of-care, first-line therapy for relapsing forms of multiple sclerosis, and IRG1 levels could modulate the IFN-β efficacy versus side effects in these patients.

Unexpectedly, we observed that IRG1 depletion in human macrophages resulted in either enhanced or reduced burdens of HMPV in cells derived from different individual donors. The Bloodbank at St. Olavs Hospital does not reveal the sex of blood donors, but we speculate that the reason for this variance in HMPV levels after IRG1 depletion may be linked to sex-specific or genetic factors. Indeed, sex differences have recently been reported for IRG1-itaconate effects in influenza virus-infected mice and in itaconate-based treatment for non-alcoholic fatty liver disease in mice [20, 43]. Interestingly, our in-house bioinformatics analysis of the human IRG1 promoter revealed binding sites for the progesterone and glucocorticoid receptors (unpublished data) known to modulate sex-dependent immune functions [59]. In line with this a recent study suggested that glucocorticoids exert anti-inflammatory effects in macrophages via mechanism involving increased itaconate [60], and IRG1 is induced by glucocorticoids in macrophage-lineage cells in a zebrafish model [61]. Glucocorticoids are indeed induced during viral infections, e.g. with influenza virus [62]. Hence, we hypothesize that IRG1 could have a hitherto unappreciated role in sex-based differences of (virus-induced) hyperinflammation.

We compared the effects of 4OI with unmodified itaconate and its isomer citraconate and show that only 4OI reduces HMPV levels and strongly suppresses IFN induction in human macrophages. These results corroborate earlier findings showing that 4OI, as opposed to itaconate, strongly reduce influenza virus and type I IFN levels [20, 22]. Regarding viral titers, 4OI has been reported to have more marked effect on influenza virus [20, 22] than we observe for HMPV, and we speculate that this could be because 4OI directly targets the Crm1-dependent nuclear transport pathway that influenza virus critically relies on for its replication [22]. Previous studies found that 4OI has distinct immunomodulatory effects compared to unmodified itaconate combined with stronger electrophilic and Nrf2 activating potential of 4OI than itaconate [15, 63]. Likewise, we found that 4OI activated Nrf2-dependent transcription to higher extents than both itaconate and citraconate, hence the Nrf2-activating capacity of 4OI was associated with its stronger reduction of HMPV levels in human macrophages. Consistent with this, we found that Nrf2 depletion enhanced HMPV levels. Citraconate was reported to exhibit immunomodulatory effects by inhibiting IRG1 and potentially acting as an electrophile and Nrf2 agonist and was found to reduce IFN responses [10]. However, similarly to our results on HMPV, citraconate did not limit influenza virus in THP-1 macrophages [10]. We observed that citraconate, as opposed to 4OI, did not enhance Nrf2-driven gene transcription in HMPV-infected human macrophages and this could potentially explain the lack of anti-HMPV effect of citraconate, since Nrf2 has been found mediate antiviral effects of 4OI on some viruses [21, 64]. In line with previous studies using other inflammatory stimuli [15, 21, 65], we show that 4OI reduces HMPV-stimulated IFN-β induction. Several mechanisms by which 4OI reduces type I IFN induction have been reported, such as 4OI-mediated NRF2 stabilization (via Keap1 inhibition, [15]) and binding of NRF2 proximal to the IFN-β gene (as shown for proinflammatory cytokines, [66]), and suppression of the STING-IRF3 pathway or IRF3 dimerization [21, 65, 67]. For HMPV infection, further studies are needed to resolve the precise mechanisms that operate in 4OI-mediated reduction of IFN-β expression. Nevertheless, excessive or prolonged type IFN levels may drive viral triggered lung disease [68, 69] and HMPV-mediated pathogenesis [55], and we therefore suggest that 4OI via reduced IFN-β levels could limit HMPV-triggered pathogenesis independently of its effect on HMPV levels, similarly to what is observed for SARS-CoV-2-mediated pathology in mice [69].

The antiviral mechanism of 4OI is incompletely understood and is likely virus-specific. Remarkably, 4OI was recently found to be proviral for an oncolytic VSV variant [8]. In influenza virus-infected cells, 4OI was found to directly target nuclear export of viral ribonucleoprotein complexes, thereby inhibiting influenza virus replication [20, 22]. For other viruses (e.g. SARS-CoV-2, HSV) Nrf2 activation by 4OI impairs viral replication, but distinct cellular mechanisms downstream of Nrf2 have not yet been identified [21, 64]. Interestingly, Nrf2 modulates expression of several key enzymes involved in lipid metabolism, and 4OI was found to rewire the lipid metabolism of LPS-treated macrophages in an Nrf2-dependent manner [47, 50]. Moreover, viruses are obligate intracellular parasites that depend on lipid biosynthesis, as e.g. recently shown for SARS-CoV-2 (a virus inhibited by 4OI; [70]). Indeed, intracellular lipid droplets were found to enhance SARS-CoV-2 levels and the production of inflammatory mediators [71], while we observed that HMPV infection enhanced neutral lipids in macrophages. We also found that levels of ACLY, an enzyme critical for fatty acid and cholesterol biosynthesis, were significantly reduced in 4OI-treated human macrophages and that inhibition of ACLY enzyme activity strongly reduced HMPV levels. This could suggest that 4OI can be exerting its antiviral activity by modulating lipid biosynthesis. If true, this could explain the broad-spectrum antiviral effects and Nrf2-dependency of 4OI. Of relevance to this, IRG1 function and itaconate species have recently been linked to lipid metabolism in atherosclerosis and human nonalcoholic steatohepatitis, and itaconate alleviated obesity and reduced expression of *de novo* lipogenesis genes like ACLY in mice [35, 43, 49, 72]. Overall, considering that 4OI reduces both HMPV and IFN-β levels in human macrophages, 4OI could have potential as an adjunct treatment to control the IFN-mediated pathogenesis observed in HMPV infections.

## Supplementary Material

ugag017_Supplemental_Files

## Data Availability

The data underlying this article will be shared on reasonable request to the corresponding author.
