# Supplementary material for: Human pneumovirus induces IFN-dependent expression of the immune-responsive gene 1 and is inhibited by 4-octyl itaconate in human macrophages
Source: NAR Mol Med. 2026 Mar 24;3(2):ugag017. doi: 10.1093/narmme/ugag017 (PMC13069674; doi:10.1093/narmme/ugag017)
Supplement: ugag017_Supplemental_Files [file ugag017_supplemental_files.zip › Source data_Spahn et al. Uncropped blots.pdf]

Figure 1A

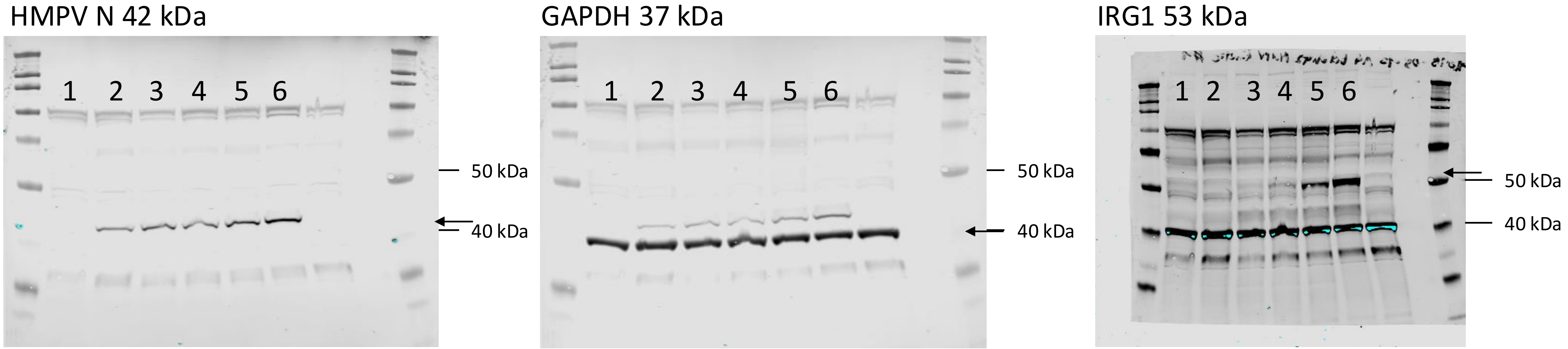

Lanes

1. Non-infected
2. HMPV 1 hour
3. HMPV 3 hours
4. HMPV 6 hours
5. HMPV 9 hours
6. HMPV 12 hours

Figure 1F

STAT1(Tyr701) 84,91 kDa

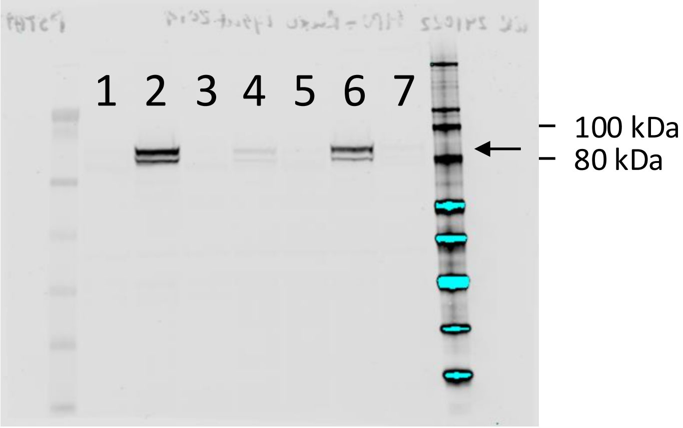

STAT1 84,91 kDa

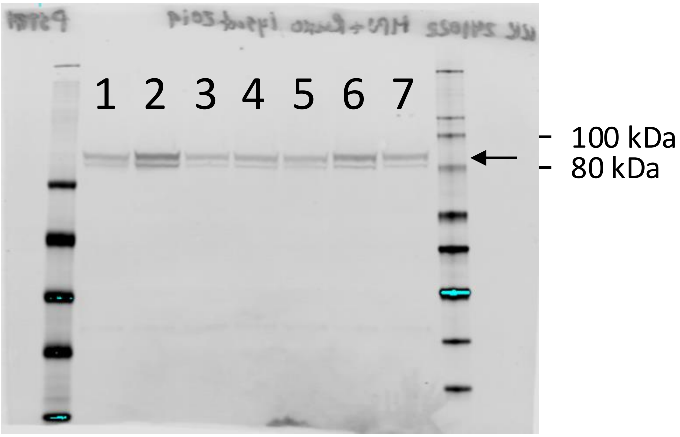

IRG1 53 kDa

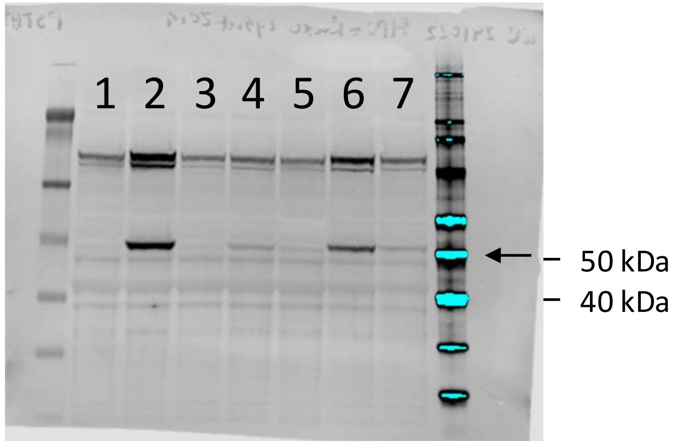

GAPDH 37 kDa

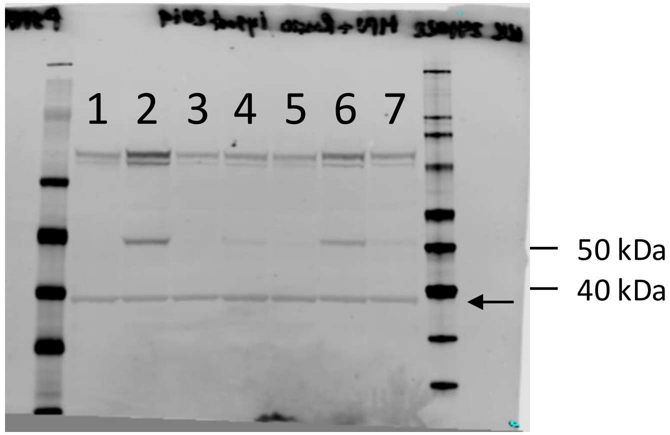

Lanes

1. Non-infected
2. HMPV 18 hours
3. S-Ruxonitilib 10 µM
4. HMPV 18 hours + S-Ruxolitinib 5 µM
5. HMPV 18 hours + S-Ruxolitinib 10 µM
6. Recombinant IFN-β
7. Recombinant IFN-β +S-Ruxolitinib 10 µM

Figure 2B

IRG1 53 kDa

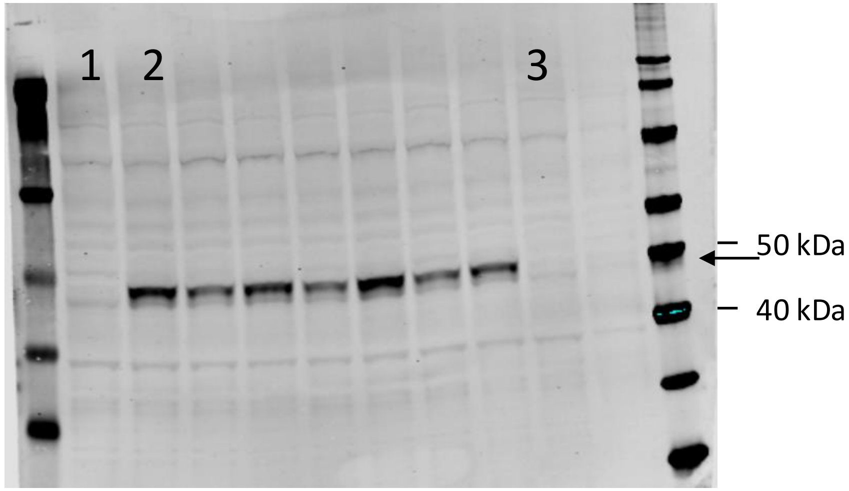

GAPDH 37 kDa

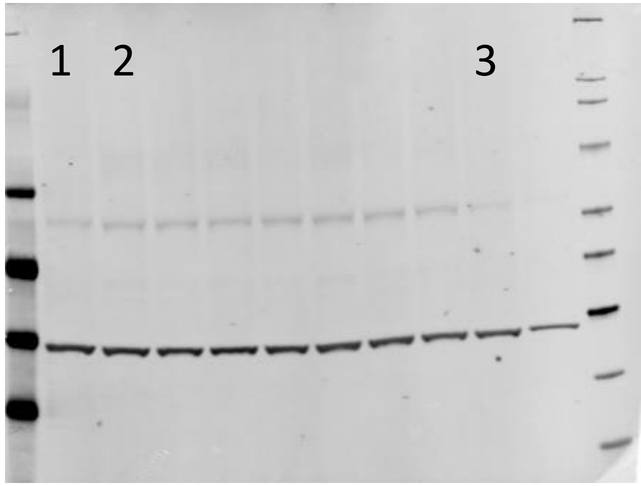

Lanes

1. Non-infected
2. HMPV 24 hours
3. BX 795 10 µM + HMPV 24 hours

Figure 2D

IRG1 53 kDa

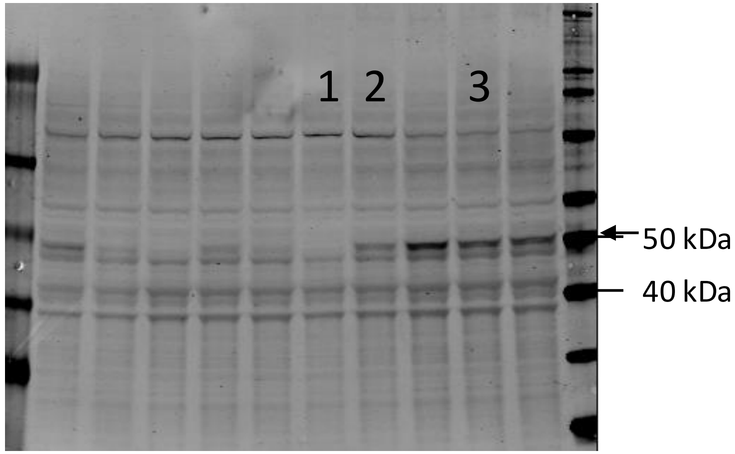

RelA/p65 65 kDa

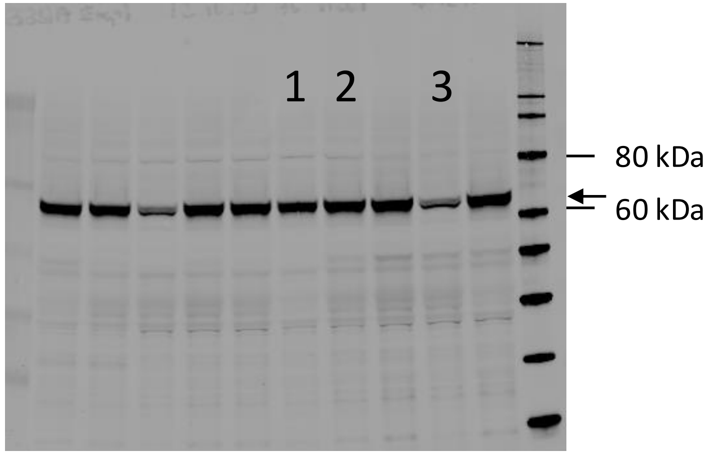

GAPDH 37 kDa

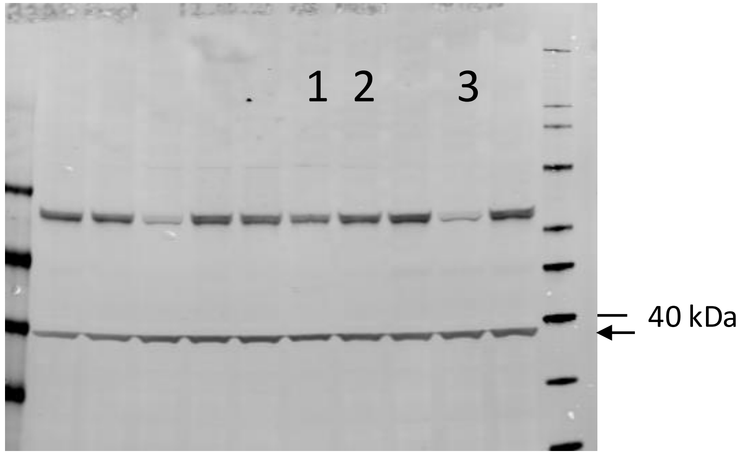

Lanes

1. siNTC medium
2. siNTC HMPV 9 hours
3. siRelA HMPV 9 hours

Figure 2G

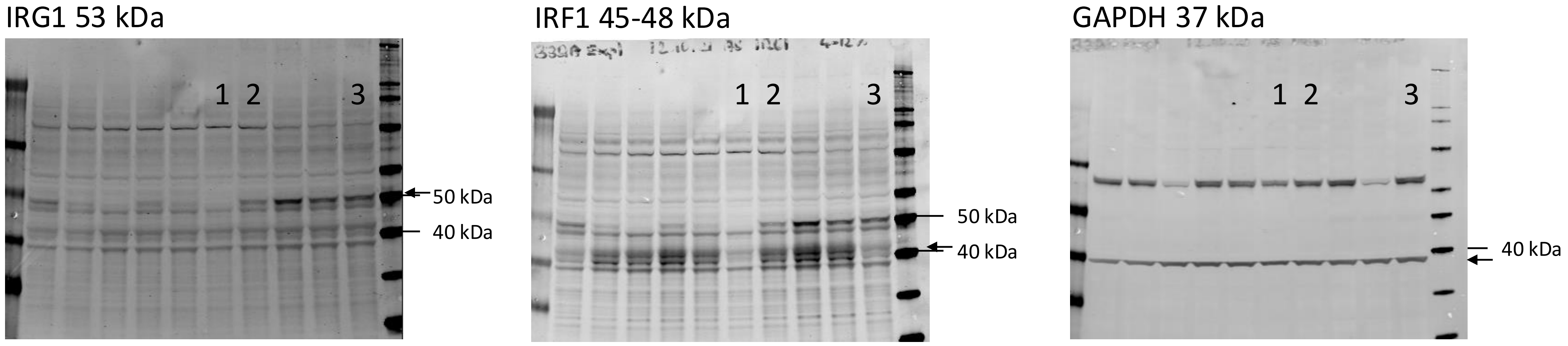

Lanes

1. siNTC medium
2. siNTC HMPV 9 hours
3. siIRF1 HMPV 9 hours

Figure 2J

IRG1 53 kDa

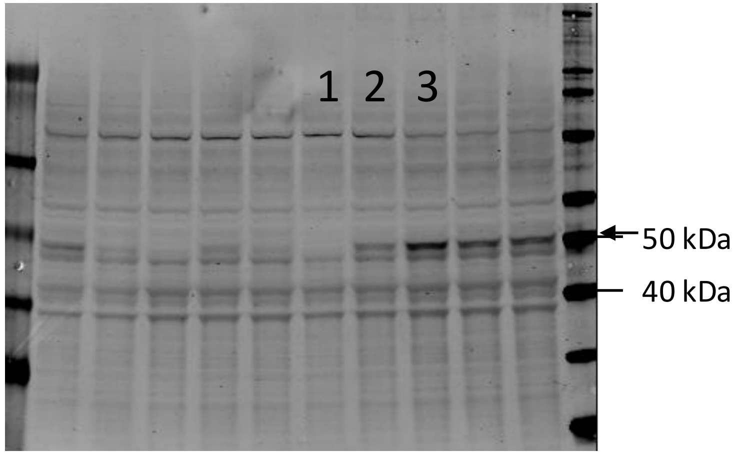

RIPK3 46-62 kDa

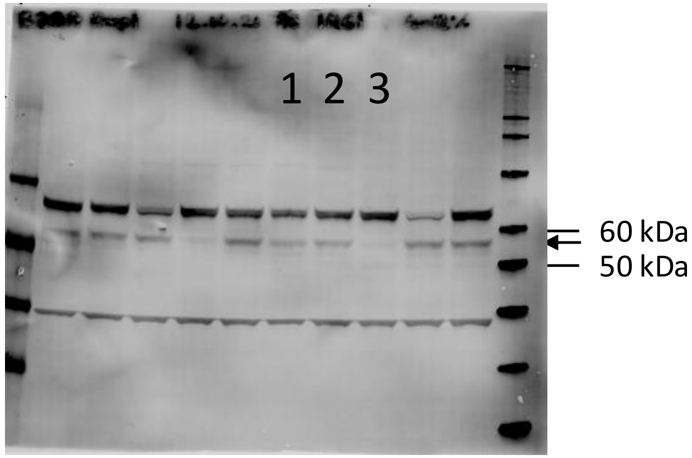

GAPDH 37 kDa

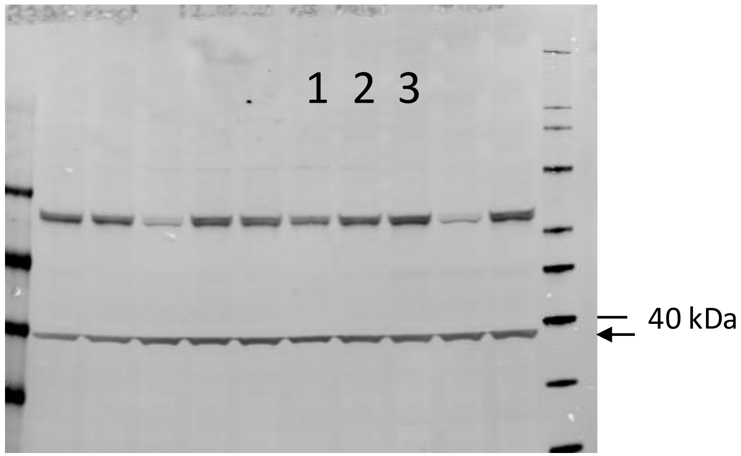

Lanes

1. siNTC medium
2. siNTC HMPV 9 hours
3. siRIPK3 HMPV 9 hours

Figure 3B

HMPV N 42 kDa

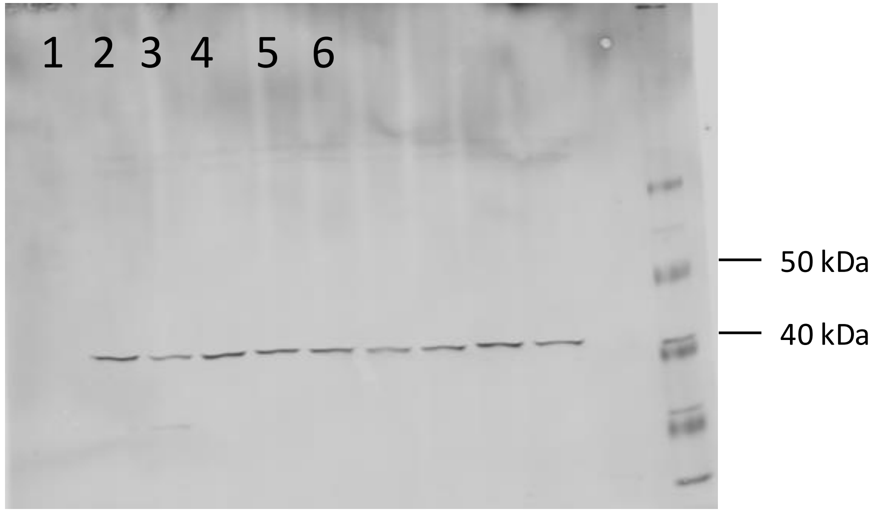

GAPDH 37 kDa

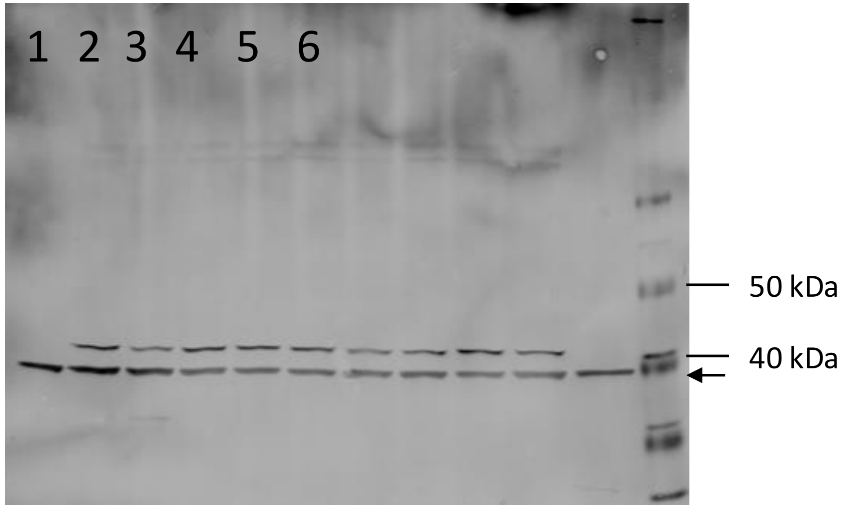

Lanes

1. Medium
2. 4-octyl itaconate 100 µM + HMPV 24 hours
3. 4-octyl itaconate 250 µM + HMPV 24 hours
4. DMSO 100 µM + HMPV 24 hours
5. DMSO 250 µM + HMPV 24 hours
6. HMPV 24 hours

Figure 3E

HMPV N 42 kDa

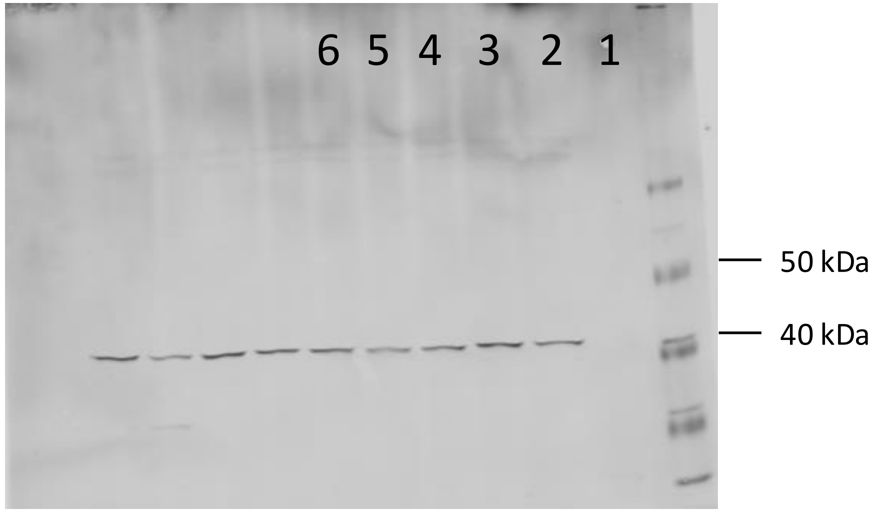

GAPDH 37 kDa

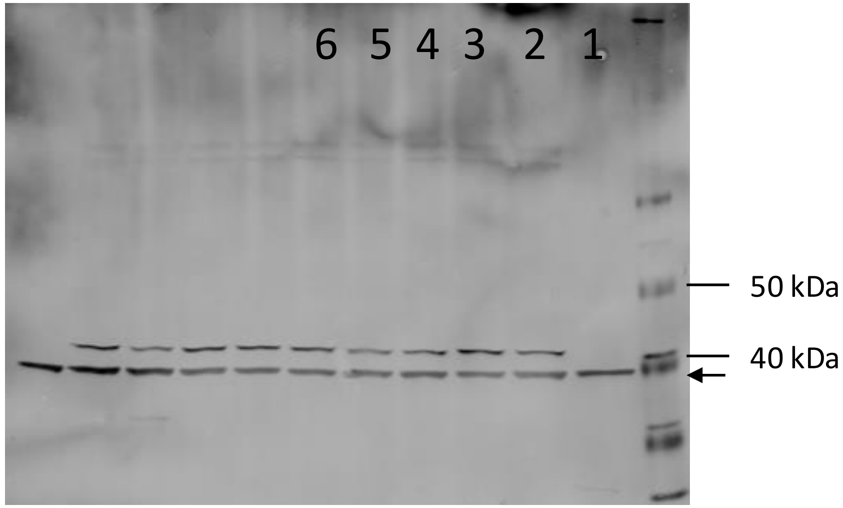

Lanes

1. Medium
2. Itaconate 0.5 mM + HMPV 24 hours
3. Itaconate 5 mM + HMPV 24 hours
4. Itaconate 10 mM + HMPV 24 hours
5. Itaconate 20 mM + HMPV 24 hours
6. HMPV 24 hours

Figure 3G

HMPV N 42 kDa

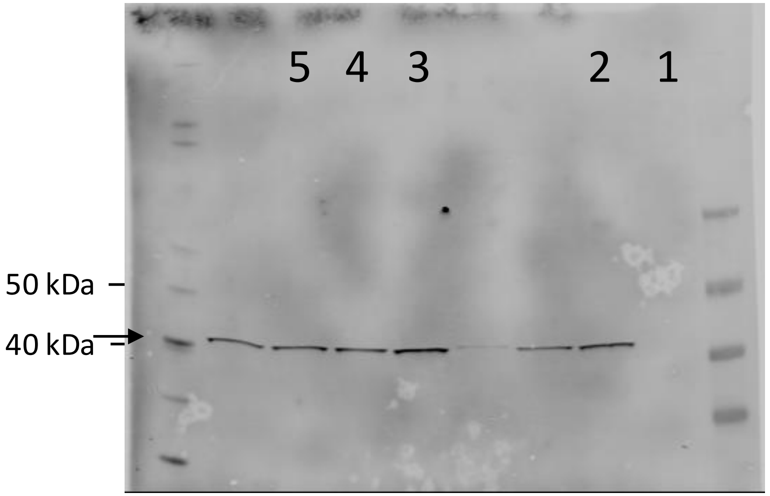

GAPDH 37 kDa

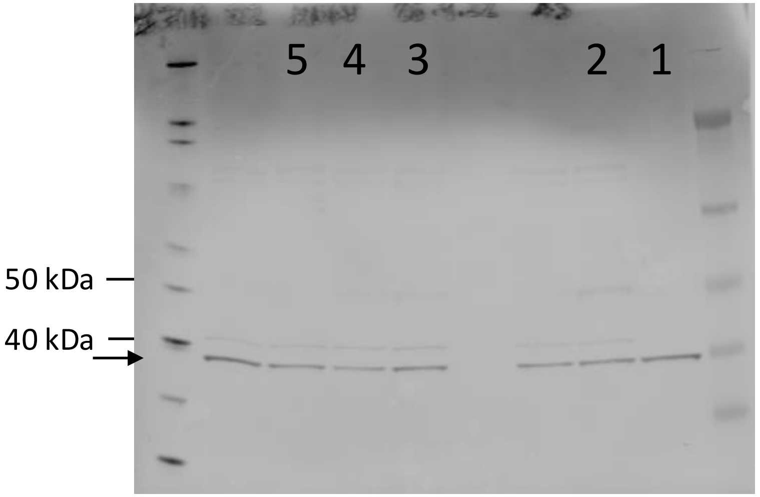

Lanes

1. Medium
2. HMPV 24 hours
3. Citraconate 10 mM + HMPV 24 hours
4. Citraconate 20 mM + HMPV 24 hours
5. Citraconate 50 mM + HMPV 24 hours

Figure 4A

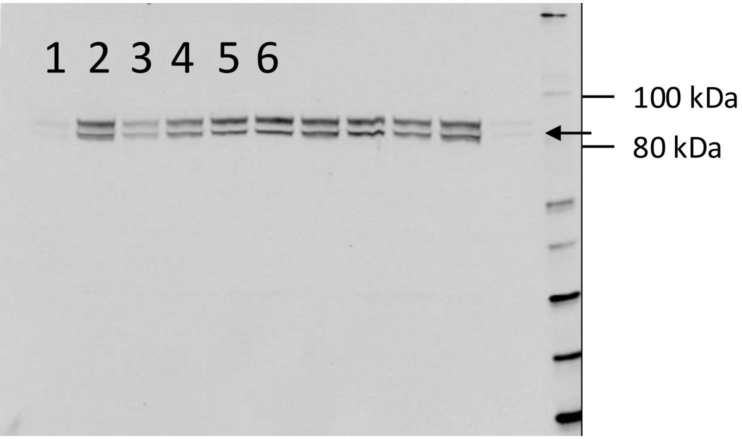

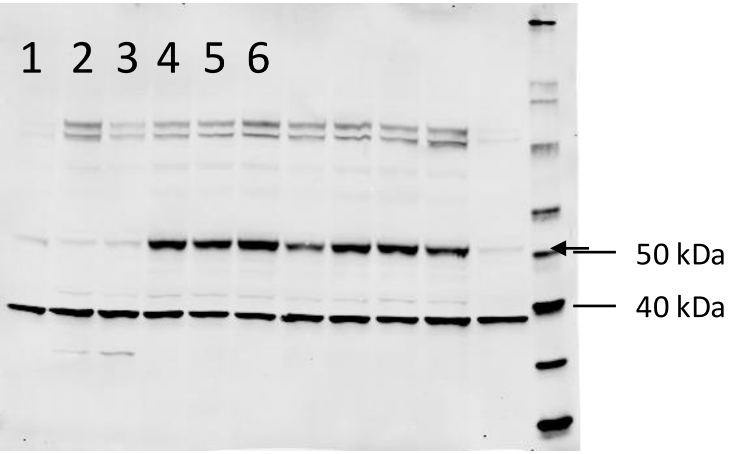

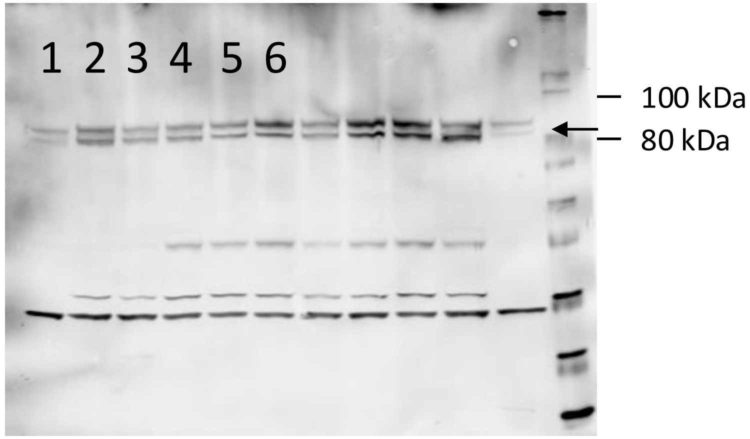

GAPDH 37 kDa

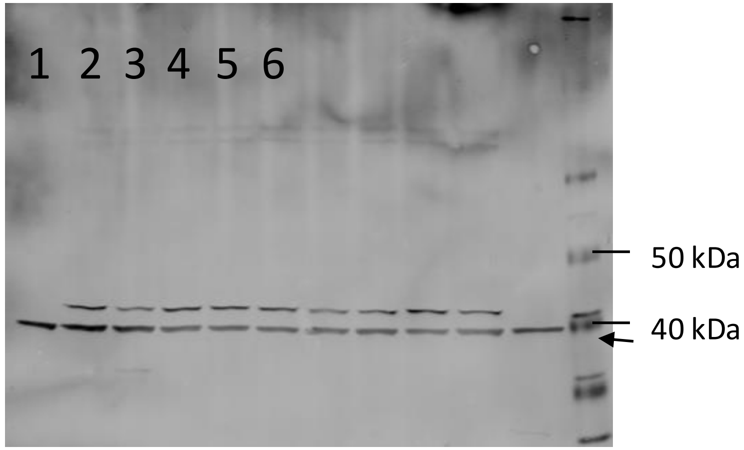

Lanes

1. Medium
2. 4-octyl itaconate 100 μM + HMPV 24 hours
3. 4-octyl itaconate 250 μM + HMPV 24 hours
4. DMSO 100 μM + HMPV 24 hours
5. DMSO 250 μM + HMPV 24 hours
6. HMPV 24 hours

Figure 4B

STAT1(Tyr 701) 91, 84 kDa

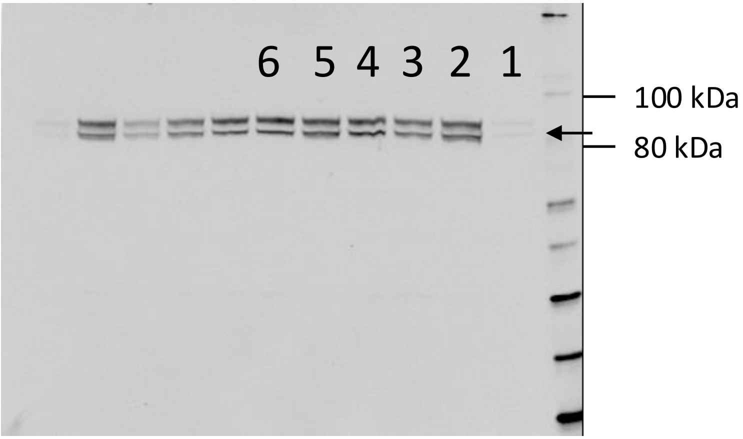

IRG1 53 kDa

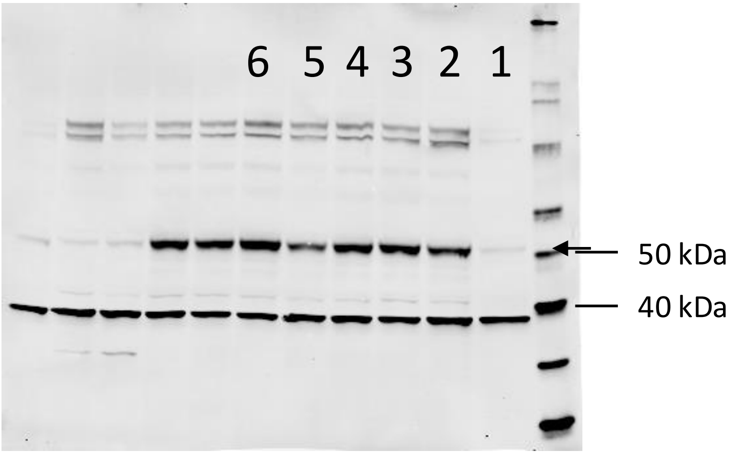

STAT1 91, 84 kDa

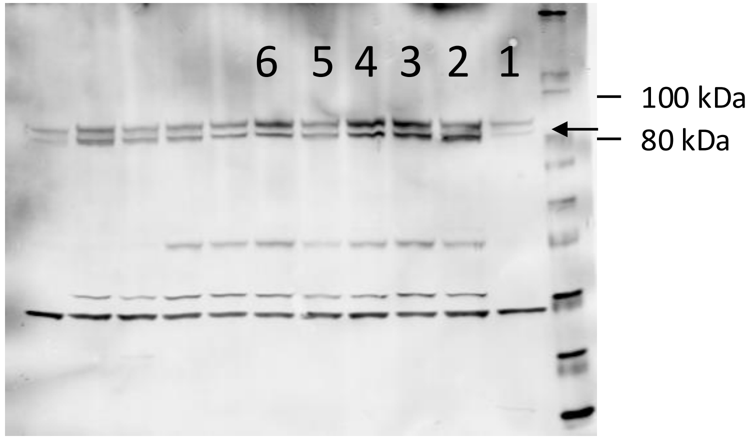

GAPDH 37 kDa

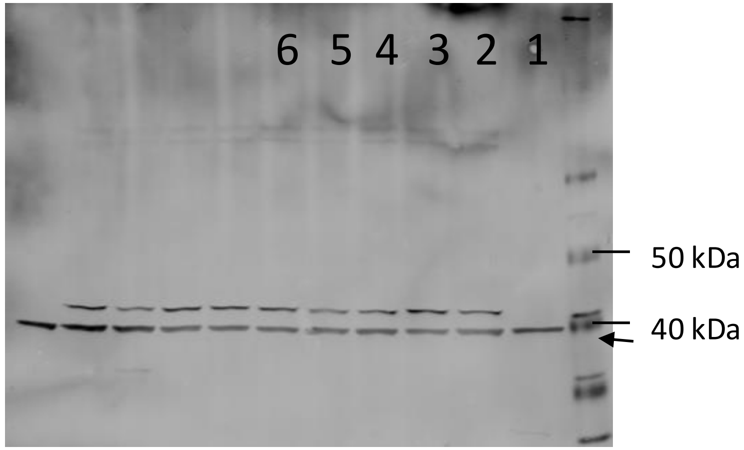

Lanes

1. Medium
2. Itaconate 0.5 mM + HMPV 24 hours
3. Itaconate 5 mM + HMPV 24 hours
4. Itaconate 10 mM + HMPV 24 hours
5. Itaconate 20 mM + HMPV 24 hours
6. HMPV 24 hours

Figure 4C

STAT1(Tyr 701) 91, 84 kDa

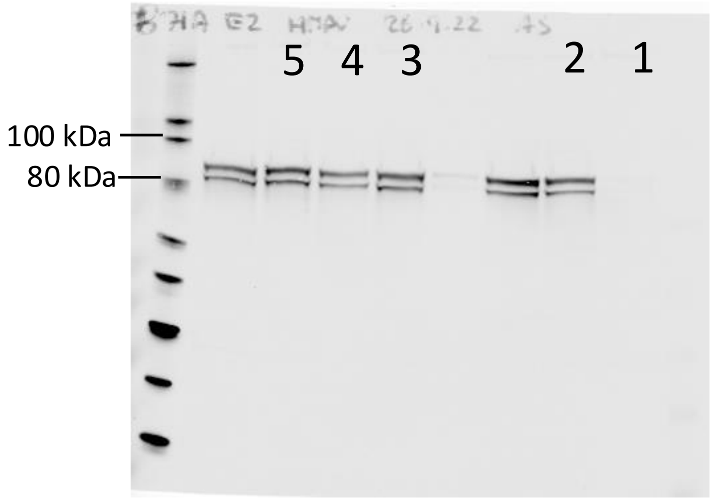

IRG1 53 kDa

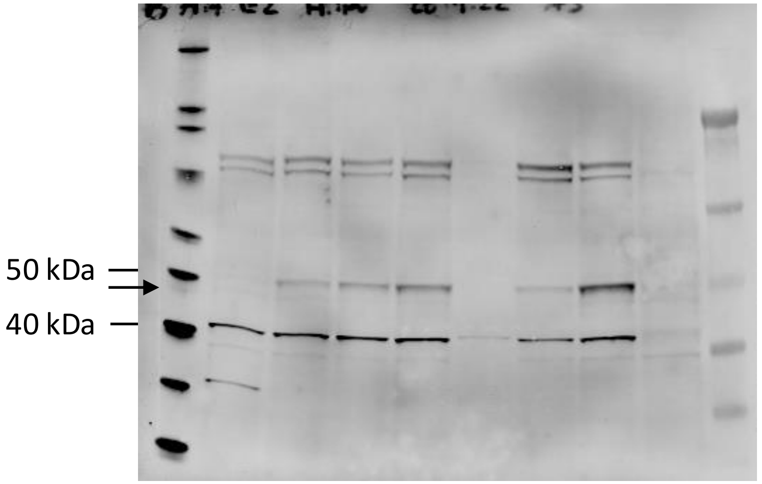

GAPDH 37 kDa

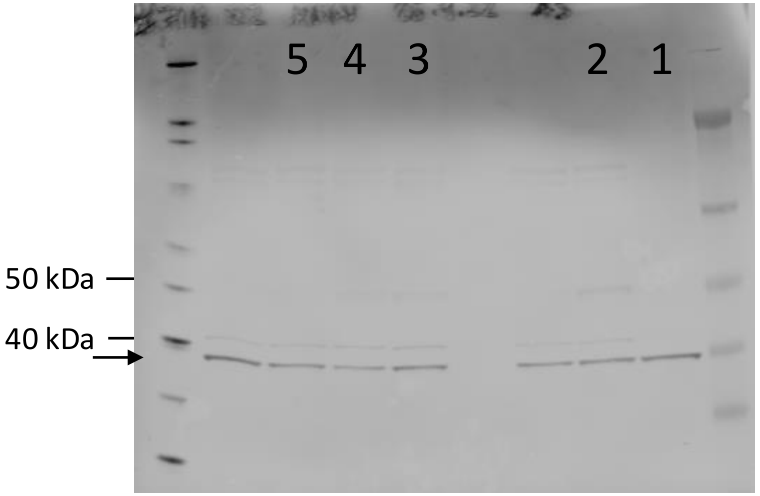

STAT1 91, 84 kDa

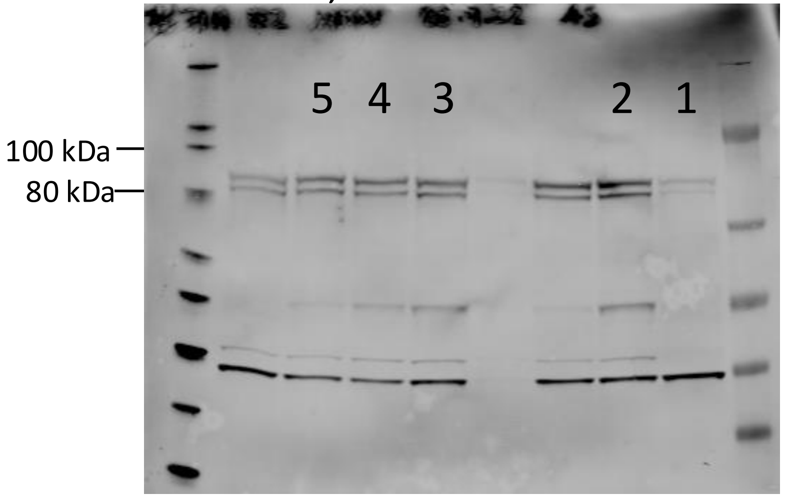

Lanes

1. Medium
2. HMPV 24 hours
3. Citraconate 10 mM + HMPV 24 hours
4. Citraconate 20 mM + HMPV 24 hours
5. Citraconate 50 mM + HMPV 24 hours

Figure 5A

HO1 32 kDa

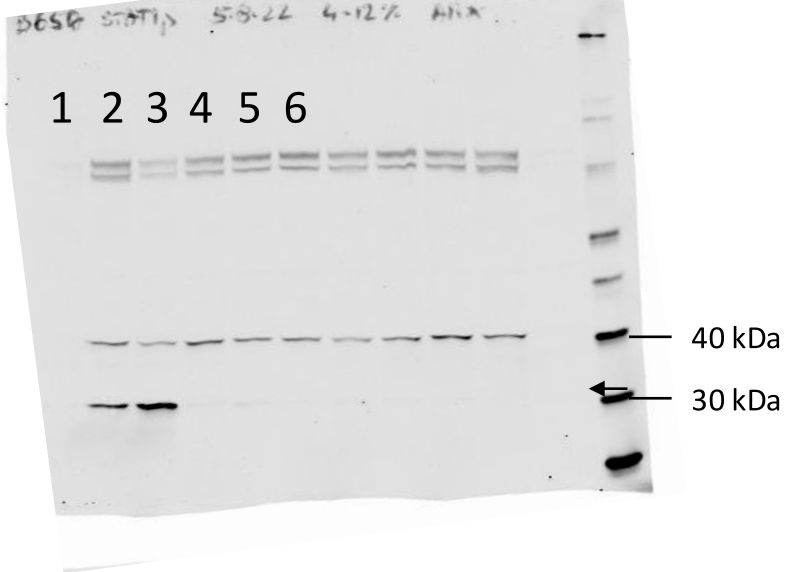

GAPDH 37 kDa

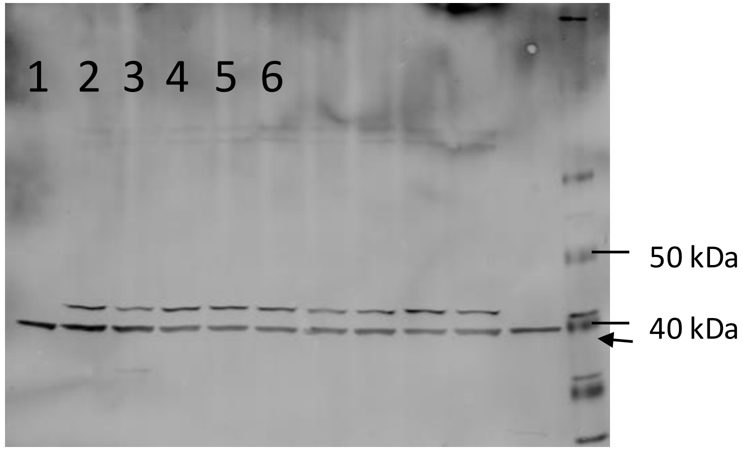

Lanes

1. Medium
2. 4-octyl itaconate 100 µM + HMPV 24 hours
3. 4-octyl itaconate 250 µM + HMPV 24 hours
4. DMSO 100 µM + HMPV 24 hours
5. DMSO 250 µM + HMPV 24 hours
6. HMPV 24 hours

Figure 5B

HO1 32 kDa

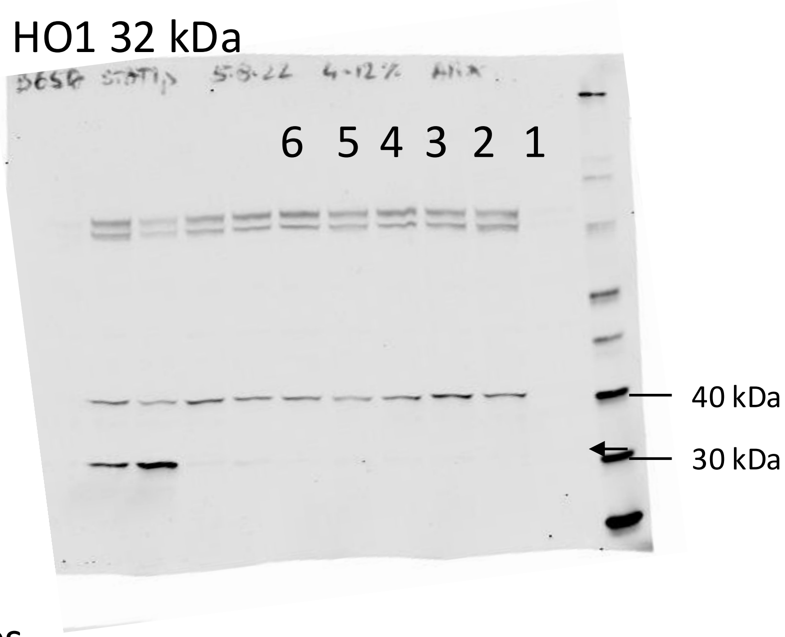

GAPDH 37 kDa

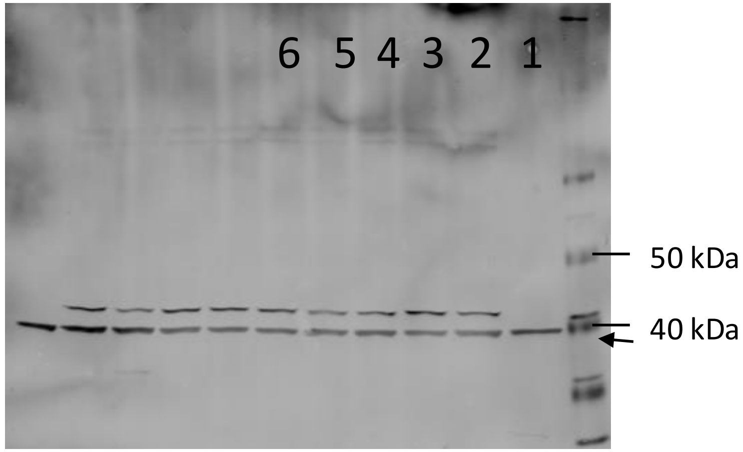

Lanes
1. Medium
2. Itaconate 0.5 mM + HMPV 24 hours
3. Itaconate 5 mM + HMPV 24 hours
4. Itaconate 10 mM + HMPV 24 hours
5. Itaconate 20 mM + HMPV 24 hours
6. HMPV 24 hours

Figure 5C

HO1 32 kDa

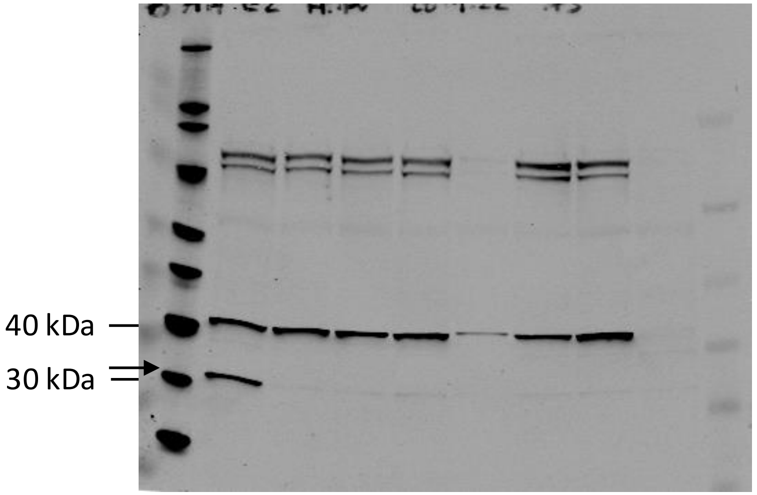

GAPDH 37 kDa

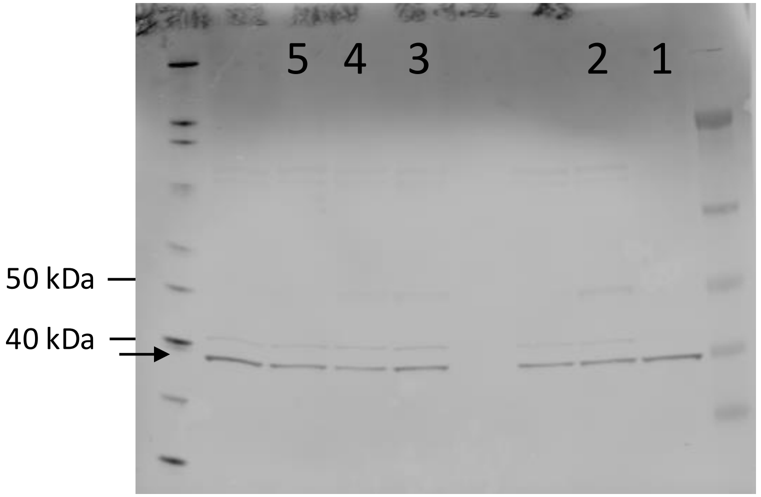

Lanes

1. Medium
2. HMPV 24 hours
3. Citraconate 10 mM + HMPV 24 hours
4. Citraconate 20 mM + HMPV 24 hours
5. Citraconate 50 mM + HMPV 24 hours

Figure 5D

Nrf2 95 kDa

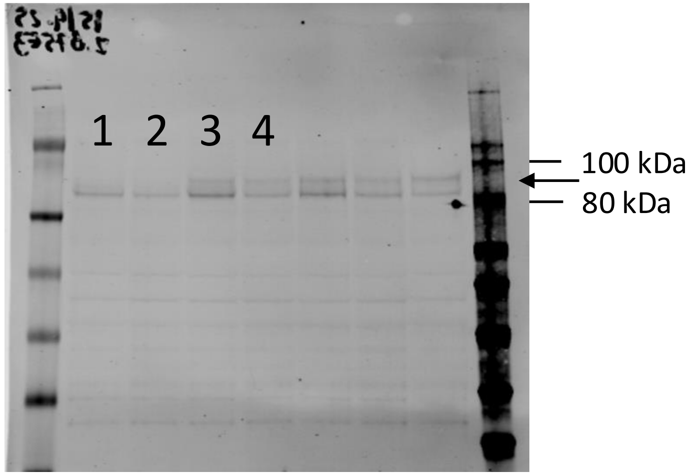

HMPV N 42 kDa

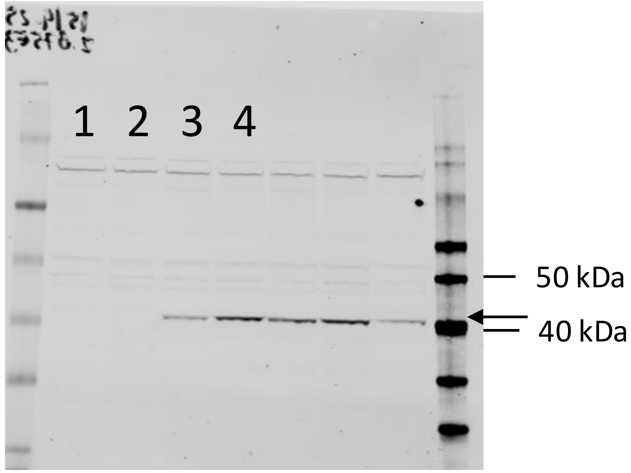

GAPDH 37 kDa

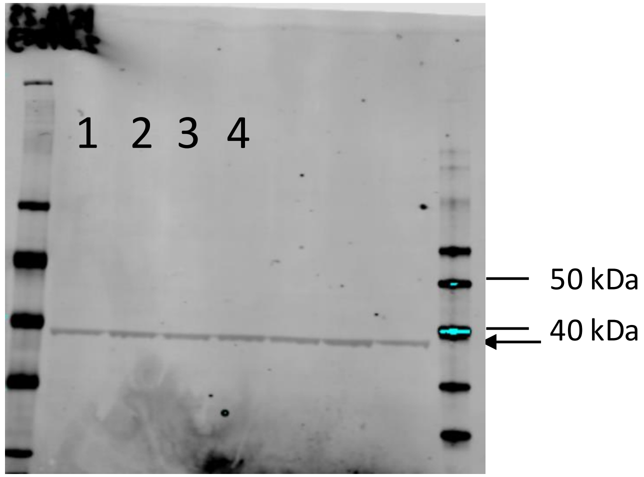

Lanes

1. siNTC medium
2. siNrf2 medium
3. siNTC HMPV 24 hours
4. siNrf2 HMPV 24 hours

Figure 6B

HMPV N 42 kDa

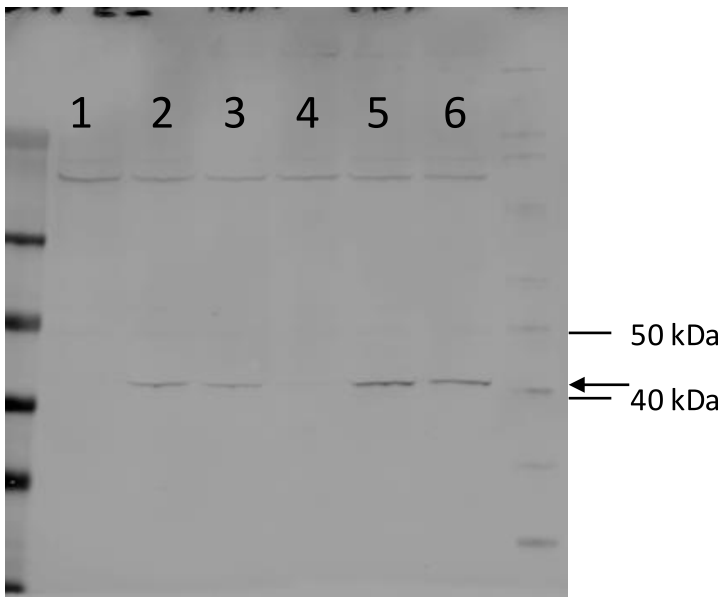

GAPDH 37 kDa

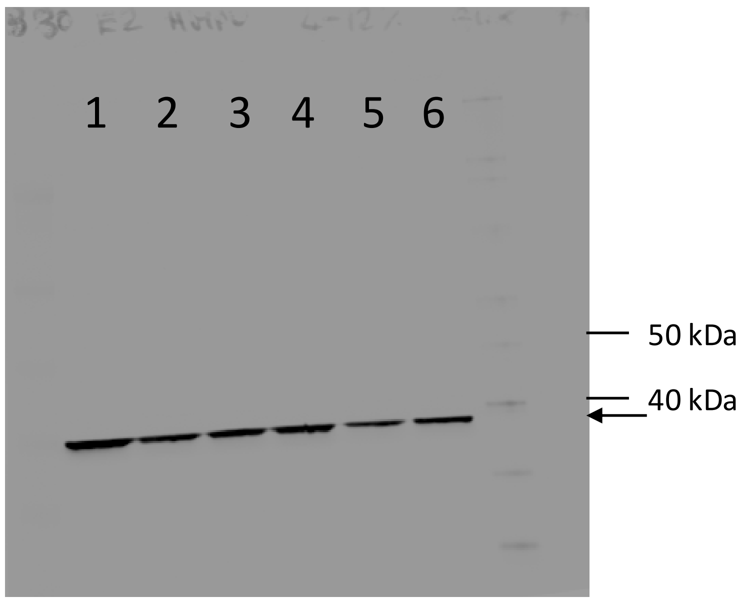

Lanes

1. Medium
2. HMPV 24 hours
3. BMS-303141 10 µM + HMPV 24 hours
4. BMS-303141 20 µM + HMPV 24 hours
5. DMSO 10 µM + HMPV 24 hours
6. DMSO 20 µM + HMPV 24 hours
