# Supplementary material for: Human pneumovirus induces IFN-dependent expression of the immune-responsive gene 1 and is inhibited by 4-octyl itaconate in human macrophages
Source: NAR Mol Med. 2026 Mar 24;3(2):ugag017. doi: 10.1093/narmme/ugag017 (PMC13069674; doi:10.1093/narmme/ugag017)
Supplement: ugag017_Supplemental_Files [file ugag017_supplemental_files.zip › Supplementary data Spahn et al. .pdf]

Supplementary data

Human pneumovirus induces IFN-dependent expression of the immune responsive gene 1 and is inhibited by 4-octyl itaconate in human macrophages

Spahn et al.

Supplementary Figures S1, S2 and Table S1.

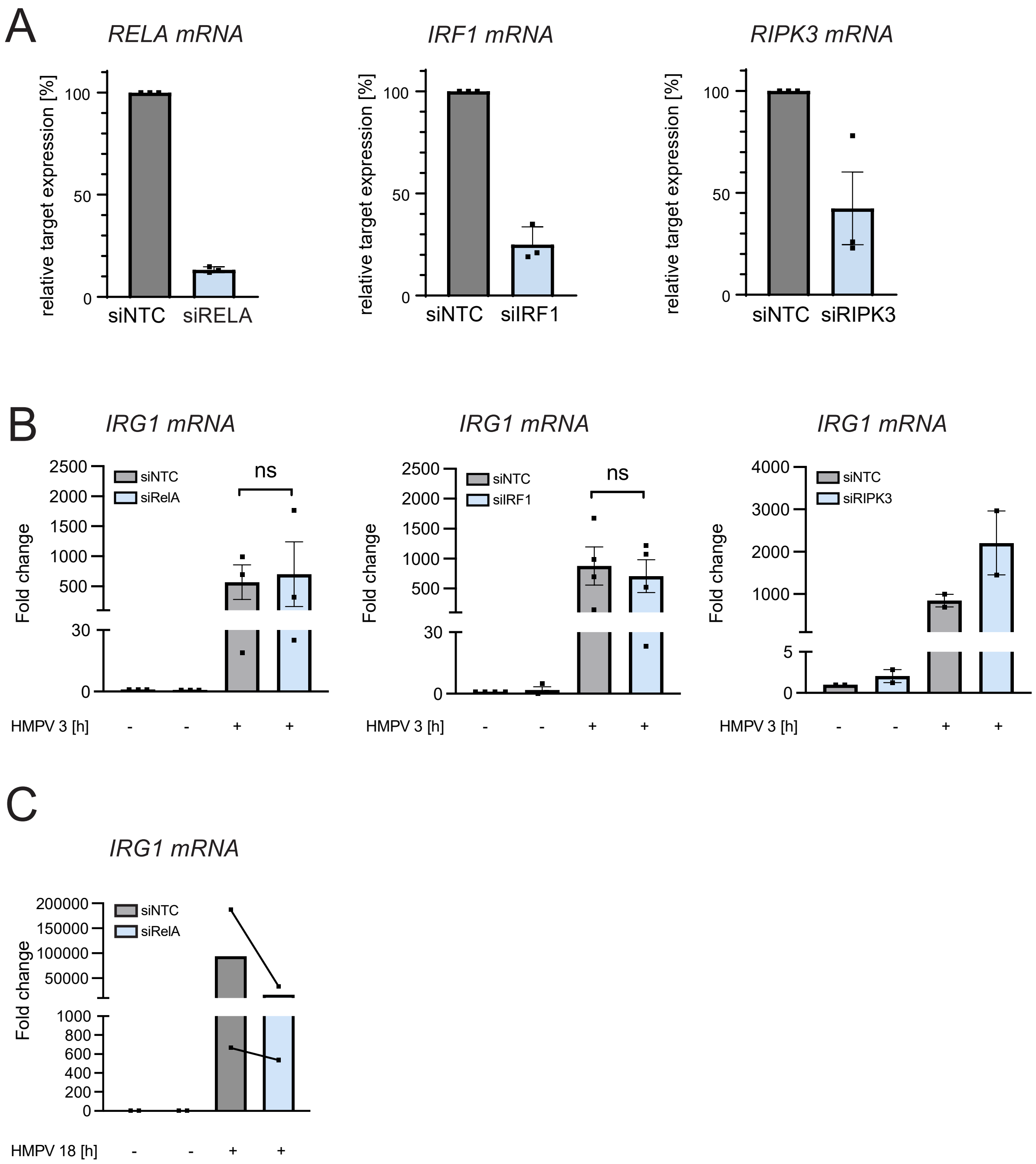

Figure S1. Transfection of human MDMs with si*RELA*, si*IRF1* and si*RIPK3*.
(A-C) MDMs were transfected with siRNAs targeting *RELA, IRF1* or *RIPK3* before infection with HMPV and analysis of mRNA expression by qRT-PCR analysis.
(A) Knockdown efficiency of *RELA, IRF1* and *RIPK3* after siRNA-mediated transfection.
(B) IRG1 mRNA levels after siRNA-mediated transfection of *RELA, IRF1* and *RIPK3* and 3 h HMPV infection.
(C) IRG1 mRNA levels after si*RELA*-transfection and 18 h HMPV infection.

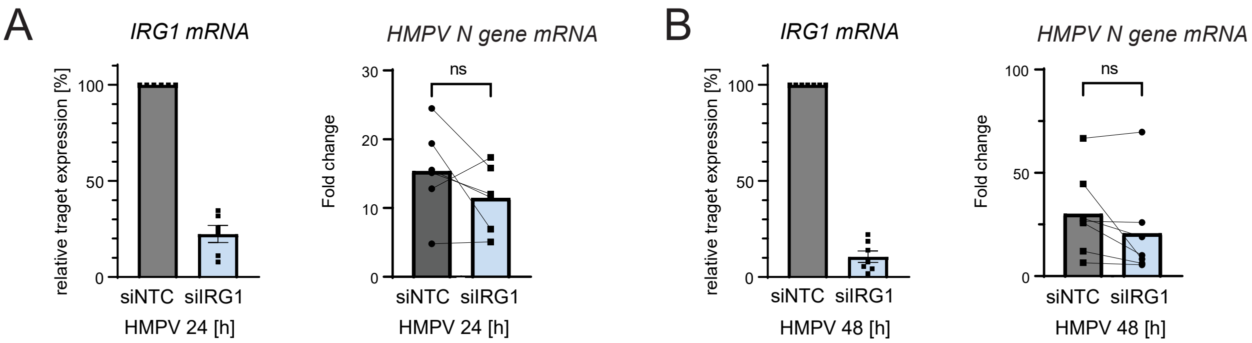

Figure S2. Deletion of IRG1 in human macrophages changes HMPV levels in a donor specific manner.

(A,B) Knockdown efficiency of IRG1 and levels of HMPV-N mRNA after siRNA- mediated transfection of human MDMs and infection with HMPV for 24 (A) or 48 (B) hrs. Levels of IRG1 of HPMV N-mRNA were determined by qRT-PCR analysis.

Primer	Forward 5'-3'	Reverse 5'-3'
hACLY	ATCCAGAGGAAGCCTACATTGC	TTTGCCAGCTCGTTGACACC
hFASN	CGCTCTGGTTCATCTGCTCT	ATGGAATCTCGGAAGCGGTC
hGAPDH	GAAGGTGAAGGTCGGAGTC	GAAGATGGTGATGGGATTTC
HMPV N-gene	CATATAAGCATGCTATATTAAAAGAGTCTC	CCTATTTCTGCAGCATATTTGTAATCAG
hHO-1	TGACCCATGACACCAAGGAC	AGTGTAAGGACCCATCGGAGA
hIFN-β	GCCGCATTGACCATCTATGAGA	GAGATCTTCAGTTTCGGAGGTAAC
hIRF1	GGCACATCCCAGTGGAAG	CCCTTCCTCATCCTCATCTGT
hIRG1	AGAAGCCCTGCCAAGGAGTCCAAA	CCAGAGCTTCTCGGCACTTTGTCG
hNQO1	CAGCTCACCGAGAGCCTAGT	GAGTGAGCCAGTACGATCAGTG
hNrf2	GAGACAGGTGAATTTCTCCCAAT	TTTGGGAATGTGGGCAAC
hRELA/p65	GTGGGGACTACGACCTGAATG	GGGGCACGATTGTCAAAGATG
hRIPK3	ATGTCGTGCGTCAAGTTATGG	CGTAGCCCCACTTCCTATGTTG
hSCD1	CTCTGCTACACTTGGGAGCC	GAGCTCCTGCTGTTATGCCC

Table S1. List of primer sequences used for quantitative RT-PCR.
